# HSP90 promotes cell glycolysis, proliferation and inhibits apoptosis by regulating PKM2 abundance via Thr-328 phosphorylation in hepatocellular carcinoma

**DOI:** 10.1186/s12943-017-0748-y

**Published:** 2017-12-20

**Authors:** Qiuran Xu, Jianfeng Tu, Changwei Dou, Jun Zhang, Liu Yang, Xin Liu, Kefeng Lei, Zhikui Liu, Yufeng Wang, Lijie Li, Hangxing Bao, Jiahui Wang, Kangsheng Tu

**Affiliations:** 1 Key Laboratory of Tumor Molecular Diagnosis and Individualized Medicine of Zhejiang Province, Zhejiang Provincial People’s Hospital (People’s Hospital of Hangzhou Medical College), Hangzhou, Zhejiang 310014 China; 2 Department of Emergency, Zhejiang Provincial People’s Hospital (People’s Hospital of Hangzhou Medical College), Hangzhou, Zhejiang 310014 China; 3 Department of Hepatobiliary Surgery, the First Affiliated Hospital of Xi’an Jiaotong University, Xi’an, Shaanxi 710061 China; 4 Department of Gastroenterology, Zhejiang Provincial People’s Hospital (People’s Hospital of Hangzhou Medical College), Hangzhou, Zhejiang 310014 China; 5 Department of Neurosurgery, Zhejiang Provincial People’s Hospital (People’s Hospital of Hangzhou Medical College), Hangzhou, Zhejiang 310014 China; 6 Department of Gynecology, Zhejiang Provincial People’s Hospital (People’s Hospital of Hangzhou Medical College), Hangzhou, Zhejiang 310014 China; 7 Department of General Surgery, Zhejiang Provincial People’s Hospital (People’s Hospital of Hangzhou Medical College), Hangzhou, Zhejiang 310014 China; 8 Zhejiang Hospital of Traditional Chinese Medical, Hangzhou, Zhejiang 310006 China; 9 School of Basic Medical Sciences, Shandong University, Jinan, Shandong 250000 China

**Keywords:** Hepatocellular carcinoma, HSP90, PKM2, Glycolysis, Growth

## Abstract

**Background:**

Heat shock protein 90 (HSP90) functions as a well-known onco-protein to regulate protein conformation, stability and degradation. Pyruvate kinase M2 (PKM2), a critical regulator of the metabolism, growth and metastasis of cancer cells, has been confirmed to be overexpressed in various human cancer including hepatocellular carcinoma (HCC). However, the molecular mechanisms underlying the oncogenic functions of HSP90 and PKM2 overexpression in HCC remain unknown.

**Methods:**

The expression of HSP90 and PKM2 in HCC specimens and cells were detected by immunoblotting and immunostaining. The interaction between HSP90 and PKM2 was confirmed by tandem affinity purification, co-immunoprecipitation and Glutathione S transferase (GST)-pulldown assay.

**Results:**

In this study, we found that HSP90 could bind to PKM2 and subsequently increased PKM2 abundance in HCC cells. Immunohistochemistry (IHC) staining showed that HSP90 level was positively correlated with PKM2 level in HCC tissues. Mechanistically, HSP90 was found to increase the phosphorylation of PKM2 at Thr-328. Protein kinase glycogen synthase kinase-3β (GSK-3β) formed a protein complex with HSP90 and PKM2, and directly mediated Thr-328 phosphorylation of PKM2 induced by HSP90. Thr-328 phosphorylation was critical for maintaining PKM2 stability and its biological functions in regulating glycolysis, mitochondria respiration, proliferation and apoptosis. Functionally, we found that HSP90 promoted the glycolysis and proliferation and inhibited apoptosis of HCC cells in a PKM2 dependent manner. In vivo experiments disclosed that PKM2 was required for the promoting effects of HSP90 on the growth of HCC cells in mice. Furthermore, we demonstrated that positive expression of HSP90 and PKM2 was correlated with poor clinicopathological features including high alpha fetoprotein (AFP) level, large tumor size, portal vein tumor thrombus (PVTT) and advanced tumor-node-metastasis (TNM) stage. Furthermore, we demonstrated that positive expression of HSP90 and PKM2, and a combination of these proteins could strongly predict the poor prognosis of HCC patients.

**Conclusions:**

We suggest that HSP90 potentiates the glycolysis and proliferation, reduces the apoptosis and thus enhances the growth of HCC cells through PKM2.

**Electronic supplementary material:**

The online version of this article (10.1186/s12943-017-0748-y) contains supplementary material, which is available to authorized users.

## Background

Hepatocellular carcinoma (HCC), one of the most common malignant tumors worldwide, ranks as the third leading cause of cancer-related deaths [1]. Currently, surgical resection and liver transplantation are curative options for HCC patients [2]. Although the remarkable advance has been achieved in treatment of HCC, the long term prognosis of HCC patients remains poor [3]. The critical reasons for the unsatisfactory prognosis of HCC patients include malignant growth and systemic metastasis of HCC. However, the underlying mechanisms responsible for the growth and metastasis of HCC remain largely unknown.

Pyruvate kinase (PK), which is a rate-limiting enzyme of cellular glycolysis, exists in 4 isoforms (M1, M2, L, and R) [4]. Among these four isoforms, PKM2 is predominantly expressed in the fetus and is replaced by PKM1 during development [5]. However, the abundance of PKM2 is dramatically increased in tumor cells, indicating an important role of PKM2 in cancer cells [6]. PKM2 has been found to be overexpressed in various cancers and acts as a critical regulator of cancer cell metabolism [7]. PKM2 leads to increased glucose uptake and lactate production and decreased O_2_ consumption of cancer cells. PKM2 also promotes the anabolic metabolism of glucose for macromolecular biosynthesis and enhances the growth of cancer cells [4]. Additionally, PKM2 acts as a transcriptional coactivator to promote the gene transcription in cancer cells [8, 9]. Previous study in ovarian cancer cells found that. CHIP (carboxyl terminus of Hsc70-interacting protein), a U-box E3 ligase, was found to interact with PKM2 and regulated the ubiquitination and stability of PKM2 and metabolism [10]. Ubiquitination of PKM2 mediated by Parkin, another ubiquitin E3 ligase, led to decreased enzymatic activity of PKM2 [11]. Moreover, Ubiquitin-specific protease 20, one of deubiquitinating enzymes (DUBs), was found to bind with PKM2 and regulated PKM2 ubiquitination [12]. Therefore, the interaction between PKM2 and its binding proteins could alter the ubiquitination status of PKM2, and was critical for maintaining the expression level and functions of PKM2. Our previous study found that the expression of PKM2 was significantly increased in HCC [13]. However, the underlying mechanisms responsible for the aberrant expression of PKM2 in HCC remain unknown.

The heat shock proteins (HSPs), a group of highly conserved molecular chaperones, play various roles in cell function including protein folding, the assembly of protein complex and protein degradation [14]. HSP90, a member of HSPs family, has been found to play critical roles in regulating the proliferation, apoptosis and metastasis of tumor cells [15]. HSP90 can regulate the conformation, stability and function of various oncogenic proteins to participate in the development and progression of human cancer [16]. Our previous studies showed that HSP90 was overexpressed in HCC and was associated with unfavorable clinicopathological features and poor prognosis of HCC patients [17]. However, the molecular mechanisms underlying the oncogenic functions of HSP90 in HCC progression remain unknown.

In this study, we demonstrated for the first time that HSP90 was a novel binding partner of PKM2 and increased PKM2 protein abundance in HCC by enhancing the protein stability and reducing the proteasomal degradation of PKM2. Overexpression of HSP90 led to the phosphorylation of PKM2 at Thr-328 while HSP90 knockdown reduced PKM2 Thr-328 phosphorylation. GSK-3β, a canonical protein kinase, formed a protein complex with HSP90 and PKM2, and mediated Thr-328 phosphorylation of PKM2 induced by HSP90. Thr-328 phosphorylation was critical for the protein stability and biological functions of PKM2. Functionally, we found that HSP90 promoted glycolysis and proliferation, and inhibited apoptosis of HCC cells through PKM2. Moreover, PKM2 was required for the promoting effect of HSP90 on the growth of HCC cells in vivo. Lastly, we revealed that HSP90 expression was positively correlated with PKM2 expression in HCC tissues. In all, this study demonstrated for the first time that HSP90, a newly identified binding partner of PKM2, promoted the growth of HCC by regulating Thr-328 phosphorylation and PKM2 abundance.

## Methods

### Clinical samples

One hundred HCC tissues and adjacent non-tumor tissues were collected in the Department of Hepatobiliary Surgery, the First Affiliated Hospital of Xi’an Jiaotong University. All patients did not receive any radiotherapy, chemotherapy or radiofrequency ablation before surgical resection. The clinicopathological and demographic information of the patients was described in Table 1. Patients’ tissues were collected and stored in paraformaldehyde for immunohistochemistry (IHC).

**Table 1:** Clinical correlation of HSP90 and PKM2 expression in HCC

Clinicopathological Features		n	HSP90		*P* value	PKM2		*P* value
			Positive	Negative		Positive	Negative	
Age (y)	<50	32	20	12	0.086	18	14	0.669
	≥50	68	29	39		34	34	
Sex	Male	65	31	34	0.834	35	30	0.677
	Female	35	18	17		17	18	
HBV	Absent	31	17	14	0.518	18	13	0.517
	Present	69	32	37		34	35	
Cirrhosis	Absent	21	12	9	0.466	9	12	0.462
	Present	79	37	42		43	36	
AFP level (ng/mL)	<400	22	13	9	0.338	6	16	***0.015***
	≥400	78	36	42		46	32	
Tumor size (cm)	<5	55	18	37	***0.006***	21	34	***0.003***
	≥5	45	31	14		31	14	
PVTT	Absent	36	10	26	***0.002***	11	25	***0.002***
	Present	64	39	25		41	23	
TNM stage	I + II	59	20	39	***0.005***	24	35	***0.008***
	III + IV	41	29	12		28	13	

Significant values (*P*<0.05) are in italic and bold

### Cell culture

Hep3B, Huh7, 293 T and HEK-293 cells were bought from the Institute of Biochemistry and Cell Biology, Chinese Academy of Sciences (Shanghai, China). DMEM (Dulbecco’s modified Eagle’s medium; Gibco, Grand Island, NY, USA) supplemented with 10% fetal bovine serum (FBS; Gibco), penicillin (100 units/ml, Sigma, St. Louis, MO, USA) and streptomycin (100 μg/ml, Sigma) was used for cell culture. All these cells were kept in a humidified 5% CO_2_ incubator at 37 °C. For 17-AAG (Sigma) and 17-DMAG (Sigma) treatment, the cells were cultured in serum-free DMEM for 12 h, and then were cultured in serum-free DMEM containing 17-AAG or 17-DMAG for 24 h.

### Cell transfection and retroviral transduction

One group of Control shRNA (sc-108,060) and HSP90 shRNA (sc-35,608) were obtained from Santa Cruz Biotechnology (Santa Cruz, CA, USA). Second group of HSP90 shRNA (target sequence: 5′-CCAACTCATGTCCCTCATCAT-3′) was synthesize by Genecopoeia (Guangzhou, China). These control shRNAs and HSP90-specific shRNAs were transfected into Hep3B cells using Lipofectamine 2000 (Invitrogen). Glycogen synthase kinase-3β (GSK-3β) siRNA (sc-35,527) and control siRNA (sc-37,007) were purchased from Santa Cruz Biotechnology (Santa Cruz, CA, USA) and were transfected into Huh7 cells using Lipofectamine® RNAiMAX (Thermo Fisher Scientific). Vector pLKO was purchased from Addgene (Cambridge, MA, USA) for the expression of PKM2 shRNA as previously described [13]. The retroviral vectors, pMMP-Flag-HSP90 and pMMP-HA-PKM2, were generated by cloning the respective cDNA into pMMP vector (Addgene). The packaging plasmids including pMD.MLV (1.5 μg), pVSV.G (0.5 μg) and the retroviral vectors mentioned above (2 μg) were transfected into HEK-293 T cells using Effectene Transfection reagent (Qiagen, Valencia, CA, USA). The media containing the retroviruses were collected 72 h after transfection. Viral transduction was performed by incubating the cells with the viral supernatant (25%) supplemented with Polybrene (8 μg/ml, Santa Cruz Biotechnology) overnight at 37 °C. Cells were collected for further experiments 72 h after viral transduction.

### IHC staining

IHC was performed on 5-μm-thick sections from formalin-fixed, paraffin-embedded tissue samples applied to coated slides. The procedures of IHC staining were performed as we previously described [18, 19]. The following antibodies including HSP90 (Abcam, ab13492) and PKM2 (Abcam, ab131021) were used for IHC staining of HCC tissues.

### Tandem affinity purification

Tandem affinity purification was performed as we previously described [20]. 293 T cells were transfected with SBP- and S-protein-tagged PKM2 or HSP90 and then maintained to establish the stable cell line. These cells were lysed with NETN buffer (20 mM Tris-HCl, pH 8.0, 100 mM NaCl, 1 mM EDTA, 0.5% Nonidet P-40) containing 50 mM β-glycerophosphate, 10 mM NaF, and 1 μml^−1^ each of pepstatin A and aprotinin on ice for 10 min. After removal of cell debris by centrifugation, crude cell lysates were incubated with streptavidin sepharose beads (Amersham Biosciences) for 1 h at 4 °C. The bound proteins were washed three times with NETN and then eluted with 2 mM biotin (Sigma) for 30 min twice at 4 °C. The eluates were incubated with S-protein agarose (Novagen) for 1 h at 4 °C and then washed three times with NETN. The proteins bound to S-protein agarose beads were resolved by sodium dodecyl sulphate-polyacrylamide gel electrophoresis (SDS-PAGE) and visualized by Colloidal blue or Coomassie blue staining. The identities of eluted proteins were revealed by mass spectrometry (MS) performed by the Taplin Biological Mass Spectrometry Facility at Harvard.

### Reverse transcription-quantitative polymerase chain reaction (RT-qPCR)

PCR amplifications for the quantification of PKM2, HIF-1α target genes (Glut1, Eno1, VEGF, LDHA and PDK1) and β-catenin target genes (c-Myc and CCND1) were performed using a SYBR® Premix Ex Taq™ II (Perfect Real-Time) kit (Takara Bio, Otsu, Japan) and an ABI PRISM 7300 Sequence Detection system (Applied Biosystems, Foster City, CA, USA). GAPDH was used as internal control. The primers used for qRT-PCR were listed in Additional file 1: Table S1.

### Co-Immunoprecipitation (IP) assay

The following antibodies including Flag (F1804, Sigma), Hemagglutinin (HA) (12CA5, Roche, Indianapolis, IN, USA), PKM2 (Abcam, ab131021), HSP90 (Abcam, ab13492), GSK-3β (Cell signaling, #9315), and Ser/Thr phosphorylation (Abcam, ab17464) antibodies were used in the IP assays. Detailed procedures were performed as previously described [21]. In general, total protein lysate were extracted by IP buffer. 500μg total proteins were mixed with 1 μg of the primary antibody, or IgG, and were shaken for 4 h at 4 °C. Then, the Protein G beads (GE Healthcare Life Sciences, Piscataway, NJ, USA) were added to the mixture and shaken at 4 °C overnight. The beads were then washed 3 times using IP buffer. Sample loading buffer (5X) was mixed with the beads and boiled for 10 min. The supernatant was used for western blot analysis.

### Glutathione S transferase (GST) pull-down assay

GST pull-down assay was performed in HEK293 cells due to high transfection efficiency in these cells. HA-PKM2 plasmid was transfected into HEK293 cells using Lipofectamine 2000 (Invitrogen Life Technologies) following manufacturer’s instructions. GST-HSP90 or control GST (OriGene Technologies, Inc., Rockville, MD, USA) was added into the cell lysates harvested from the cells transfected with HA-tagged PKM2. After being incubated with Glutathione beads (Sigma-Aldrich, St. Louis, MO, USA) for 2 h, the bound proteins were subjected to western blot analysis using HA antibodies. For endogenous immunoprecipitation, after being incubated with immunoglobulin G as a control, cell lysates were incubated with protein A beads (Sigma-Aldrich). The bound proteins were used for western blot with HSP90 antibody.

### Western blots

Western blots were performed as we previously described [22]. The following antibodies were used in this study including PKM2 (Abcam, ab131021), HSP90 (Abcam, ab13492), HA (12CA5, Roche), FLAG (F1804, Sigma), PKM1 (Cell signaling, #7067), Ubiquitination (Abcam, ab7780) and GAPDH (Abcam, ab9485). Cycloheximide (CHX, Abcam, ab120093), a protein synthesis inhibitor, was used to inhibit the protein synthesis. MG132 (Abcam, ab141003) was used to inhibit the proteasomal degradation.

### In vitro kinase assay

For in vitro kinase assays, WT-PKM2 or T328A-PKM2 recombinant proteins were incubated with GSK-3β (New England BioLabs) at 30 °C in a total volume of 30 μl of GSK-3β kinase assay buffer. Aliquots of reaction mixtures were analyzed by Western blots using anti- Ser/Thr phosphorylation antibody.

### Cell proliferation assays

BrdU assay was used to investigate the proliferative ability of HCC cells. HCC cells were seeded into 96-well plates at 4000 cells/well for 24 h and 5-bromodeoxy-uridine (BrdU) assay (chemiluminescent) (Roche) was used to assess cell proliferation following the protocols provided by the manufactures.

### Cell apoptosis and Caspase-3/7 activity

Annexin V-FLUOS staining kit (Roche) was used to evaluate the percentage of apoptosis. The cell samples were analyzed using BD FACSCanto II flow cytometer (Becton-Dickinson, Franklin Lakes, NJ, USA). Caspase-3/7 activity was analyzed using an Apo-ONE® Homogeneous Caspase-3/7 kit (Promega, Madison, WI, USA).

### Glucose consumption and lactate production

Cells transfected with HSP90 plasmids or PKM2 shRNAs were seeded in 6-well plates. 48 h after transfection, cells were maintained in serum-free DMEM for 24 h. The glucose and lactate levels in medium were measured using a glucose assay kit (Sigma) and a lactate assay kit (CMA, Microdialysis), respectively. The level of glucose and lactate were normalized to corresponding protein amounts (Beyotime).

### ^14^CO_2_ release assay

To evaluate ^14^CO_**2**_ release, HCC cells were incubated in glucose free medium containing 1 uCi/mL of 6-^14^C-glucose or 1-^14^C-glucose for 30 min. Phenylethylamine was added to absorb the released CO_**2**_. The radioactivity of collected ^14^CO_**2**_ was measured using by scintillation counting of phenylethylamine. The radioactivity was normalized to cell numbers.

### Cellular oxygen consumption rate

Oxygen consumption rate was evaluated using Seahorse XF24 Extracellular Flux Analyzer. Cells were plated in XF24 cell culture plates (Seahorse Bioscience) and incubated for 24 h at normal cell incubator. Cells then were equilibrated with bicarbonate-free buffered DMEM for 1 h without CO_2_ immediately before XF assay. Substrates or perturbation compounds (Oligomycin, FCCP and Antimycin/Rotenone) were added from the reagent ports to the wells at the corresponding time-points.

### Luciferase reporter assays

Cells that were transfected with wild type or T328A mutant PKM2 along with the plasmid p2.1 and pSV40-Renilla were seeded on 24-wells plates. These cells were incubated at either 20% or 1% O_2_ for 24 h. Cell lysates were subjected to the Dual-Luciferase Assay (Promega) following the manufacturer’s instructions.

### In vivo experiments

4–6 week-old female BALB/c nude mice (*n* = 12) were used to establish a xenograft tumor model. 5 × 10^6^ Huh7 cells were subcutaneously injected into the flank of each nude mouse. The width and length of the subcutaneous tumor volume were measured every 3 days and tumor volumes were calculated as follows: tumor volume = length × width × width/2. After 3 weeks, these subcutaneous tumors were removed from the nude mice and subjected to Ki67 staining (Abcam, ab15580) and TUNEL staining (Roche). All animal protocols were approved by the Institutional Animal Care and Use Committee of Xi’an Jiaotong University.

### Statistical analysis

All quantitative date were presented as the Mean ± SEM. The SPSS statistical package for Windows Version 13 (SPSS, Chicago, IL, USA) and GraphPad Prism 5 software (GraphPad Software, Inc., San Diego, CA, USA) were used for the statistical analysis including Pearson chi-square tests, two-tailed Student’s t test, a Kaplan-Meier plot, a Log-rank test, a Pearson’s correlation coefficient analysis or one-way ANOVA test. *P* < 0.05 was considered to be statistically significant.

## Results

### HSP90 is a novel binding partner for PKM2 and increases PKM2 abundance in HCC cells

**Fig. 1:**
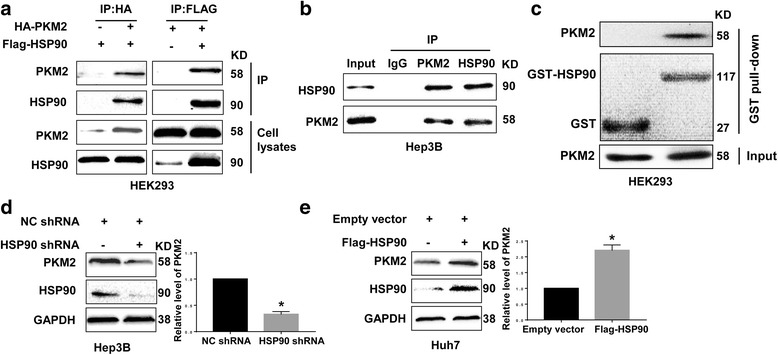
HSP90 bound to PKM2 and increased the abundance of PKM2 protein in HCC cells. **a** Flag-HSP90 and HA-PKM2 plasmids were transfected into HEK293 cells. Immunoprecipitation was performed using anti-Flag or anti-HA antibody. HSP90 and PKM2 showed an interaction between each other in HEK293 cells. **b** The interaction between endogenous HSP90 and PKM2 proteins in Hep3B cells was analyzed by Co-IP. Endogenous HSP90 and PKM2 proteins interacted with each other in Hep3B cells. **c** GST pull-down assays were performed to investigate the direct interaction between HSP90 and PKM2. **d** Hep3B cells were transfected with HSP90 shRNA or negative-control (NC) shRNA. 72 h after transfection, the levels of HSP90 and PKM2 protein in Hep3B cells were examined. Knockdown of HSP90 decreased PKM2 protein in Hep3B cells. **e** Huh7 cells were transduced with retroviruses encoding Flag-HSP90 or empty control retroviruses. 72 h after viral transduction, the levels of HSP90 and PKM2 protein in Huh7 cells were examined. Forced expression of HSP90 increased PKM2 protein abundance in Huh7 cells. *, *P* < 0.05 by t-test

**Fig. 2:**
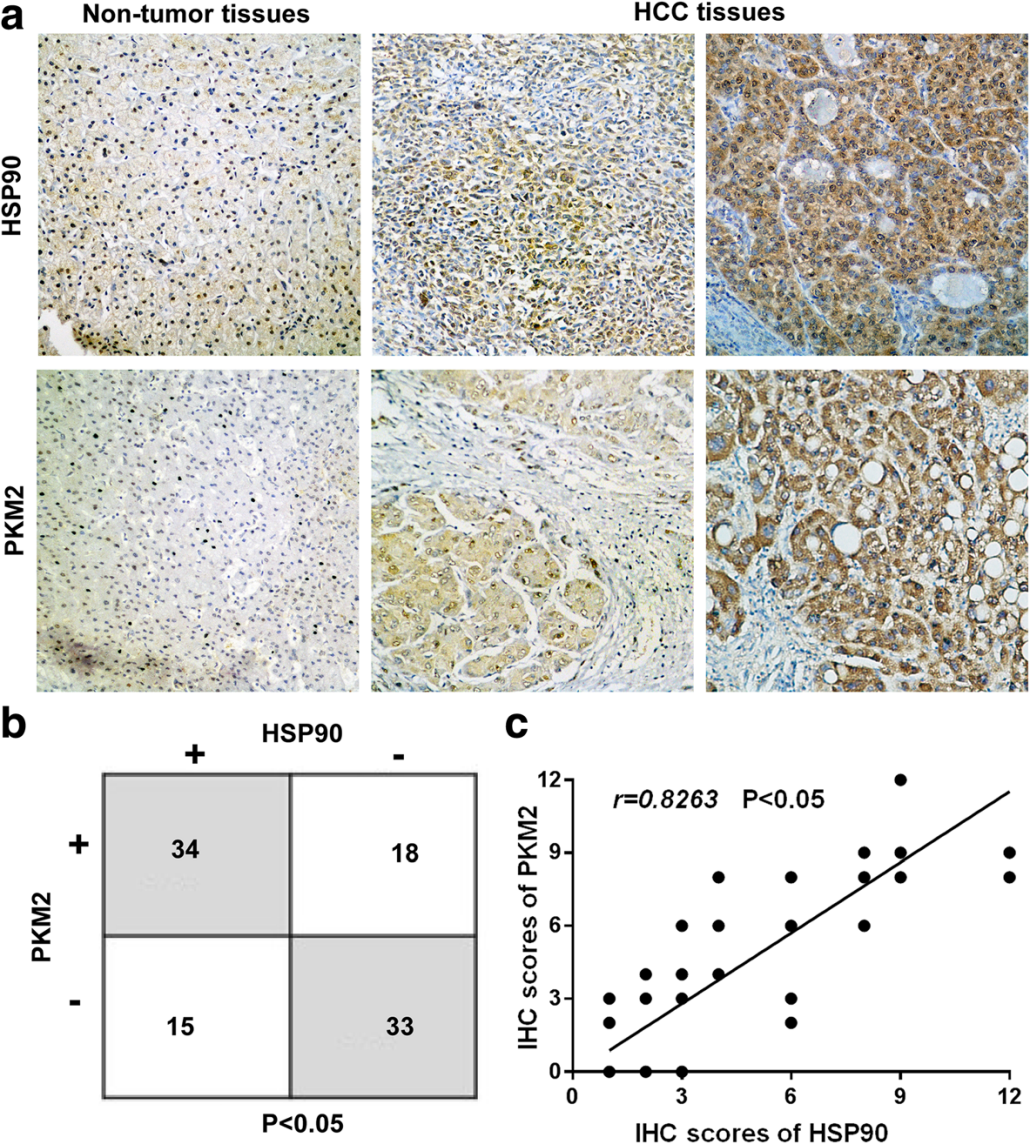
HSP90 expression was positively correlated with PKM2 expression in HCC tissues. **a** IHC staining of HSP90 and PKM2 in HCC tissues and the adjacent non-tumor tissues were performed. HSP90 and PKM2 expression in HCC tissues were increased compared with non-tumor tissues. HCC tissues with high HSP90 level showed increased PKM2 protein level compared with those with low HSP90 level. **b** Compared with tissues with negative HSP90 expression, positive rate of PKM2 was significantly increased in HCC tissues with positive HSP90 expression. **c** HSP90 IHC scores in HCC tissues were positively correlated with the IHC scores of PKM2. *, *P* < 0.05 by Chi-square test and Pearson correlation analysis

To investigate the molecular mechanisms involved in regulating PKM2 expression in HCC, we performed tandem affinity purification for PKM2 protein in 293 T cells and used MS to analyze the purified protein. The data of MS (Table 2) showed that HSP90 and PKM2 could potentially bind to each other. To confirm the potential interaction between HSP90 and PKM2 suggested by LCMS, we performed reciprocal co-IP of HA-PKM2 and Flag-HSP90 in HEK-293 cells by transfecting Flag-HSP90 and HA-PKM2 plasmids into HEK293 cells. As shown in Fig. 1a, co-IP demonstrated that HSP90 and PKM2 could interact with each other in HEK293 cells. Moreover, IP for endogenous protein also showed that HSP90 could bind to PKM2 in Hep3B cells (Fig. 1b). Furthermore, GST-pulldown assay showed that HSP90 could directly interact with PKM2 (Fig. 1c). Next, we investigated whether the interaction between HSP90 and PKM2 affected PKM2 abundance in HCC cells. To this end, Hep3B cells were transfected with two different HSP90 shRNAs which effectively reduced HSP90 level in Hep3B cells and subsequently led to decreased level of PKM2 protein (Fig. 1d and Additional File 2: Figure S1). Similarly, 17-AAG and 17-DMAG, two kinds of HSP90 inhibitors, significantly reduced PKM2 level in Hep3B cells (Additional File 3: Figure S2). On the other hand, overexpression of HSP90 in Huh7 cells significantly increased PKM2 level (Fig. 1e). To further confirm that HSP90 could increase PKM2 abundance in HCC, we performed IHC staining for HCC tissues. As shown in Fig. 2a, both HSP90 and PKM2 protein levels were significantly increased compared with non-tumor tissues. Moreover, HCC tissues with high level of HSP90 had increased expression of PKM2 protein compared with those with low level of HSP90. Positive staining of PKM2 was detected in 18 of 51 (35.29%) HCC samples with negative HSP90 expression, whereas 34 of 49 (69.39%) HCC specimens with positive HSP90 expression showed PKM2 signal (*P* < 0.05, Fig. 2b). Furthermore, Spearman correlation analysis indicated that HSP90 IHC scores were positively correlated with PKM2 IHC scores in HCC tissues (*r* = 0.8263, *P* < 0.05, Fig. 2c). Taken together, these data demonstrate that HSP90 interacts with PKM2 and increases PKM2 abundance in HCC.

**Table 2:** Purification of PKM2- and HSP90-associated proteins

PKM2 purification			HSP90 purification		
Protein	Peptides	Coverage (%)	Protein	Peptides	Coverage (%)
PKM2	62	51.24	HSP90	54	50.71
HSP90	35	28.92	PKM2	21	23.58

### HSP90 inhibits the proteasome degradation of PKM2 in HCC cells

**Fig. 3:**
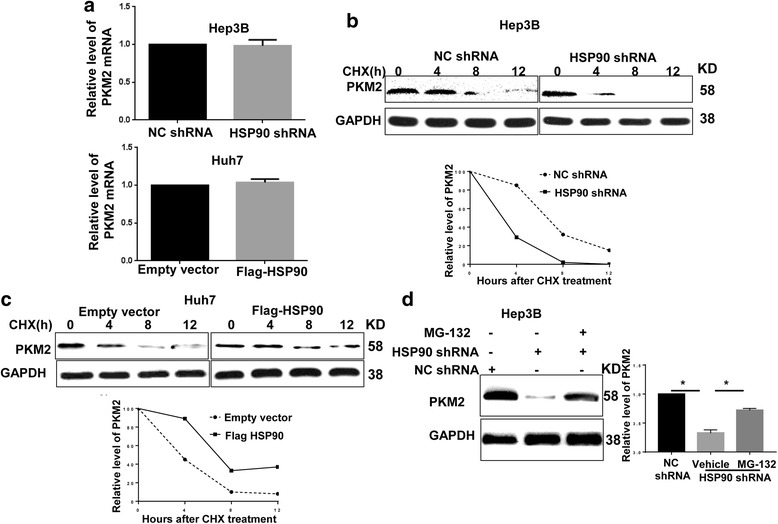
HSP90 regulated the stability of the PKM2 protein in HCC cells. **a** Huh7 cells were transfected with empty vector or Flag-HSP90 vector. Hep3B cells were transfected with negative control (NC) shRNA or HSP90 shRNA. qRT-PCR demonstrated that neither HSP90 overexpression or knockdown changed the mRNA level of PKM2. **b** & **c** The protein half-life of PKM2 in Hep3B cells was analyzed following treatment with cycloheximide (CHX). The PKM2 turnover rate was lower in Hep3B cells with HSP90 knockdown and it was higher in HSP90 overexpressing Huh7 cells. **d** MG-132 was used to inhibit the proteasomal degradation in Hep3B cells. MG-132 treatment reversed the downregulation of PKM2 protein induced by HSP90 knockdown. *, *P* < 0.05 by one way ANOVA test

To identify the mechanism by which HSP90 increases PKM2 abundance in HCC cells, we performed qRT-PCR in HCC cells to check whether HSP90 enhanced PKM2 level by promoting its transcription. The data of qRT-PCR showed that neither HSP90 knockdown nor HSP90 overexpression affected the mRNA level of PKM2 in HCC cells (Fig. 3a). These data eliminate the possibility that HSP90 increases PKM2 level in HCC cells through promoting the transcriptional activation of PKM2. Then, we investigated whether HSP90 increased PKM2 level in HCC cells by regulating its protein stability. We used CHX (Cycloheximide, a protein synthesis inhibitor) chase assays to evaluate the protein stability of PKM2. As shown in Fig. 3b, knockdown of HSP90 in Hep3B cells substantially decreased the half time of PKM2 protein. In contrary, overexpression of HSP90 in Huh7 cells remarkably increased the half time of PKM protein (Fig. 3c). Furthermore, MG132, the proteasome inhibitor, rescued the decrease of PKM2 induced by HSP90 knockdown (P < 0.05, Fig. 3d). Western blot for detergent insoluble protein confirmed that MG132 restored PKM2 protein level in the detergent-insoluble fraction (Additional File 4: Figure S3). These data indicate that HSP90 increases PKM2 level in HCC cells by reducing the proteasome degradation and enhancing the protein stability of PKM2.

### HSP90 enhances PKM2 stability by inducing the phosphorylation of PKM2 at Thr-328

**Fig. 4:**
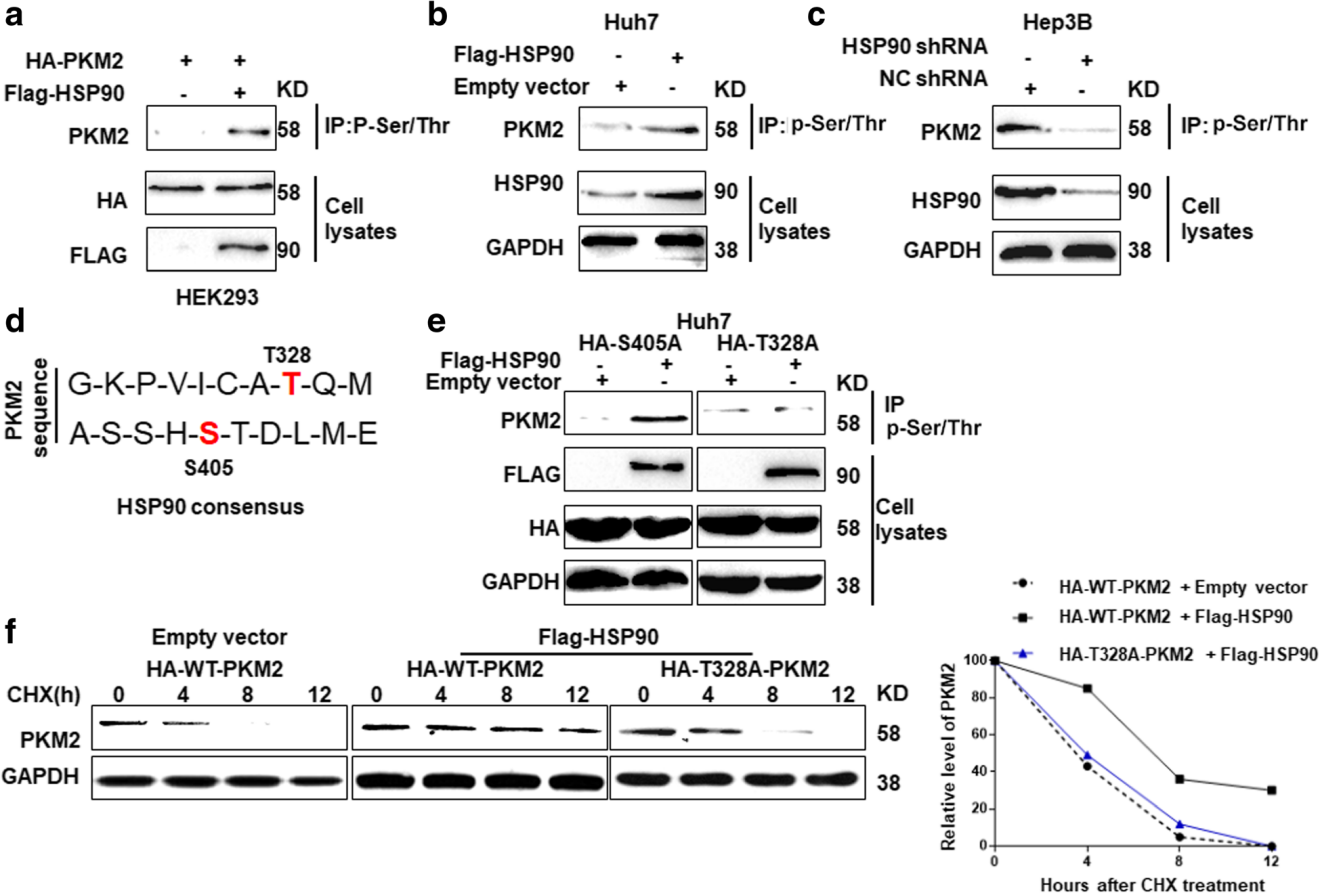
HSP90 increased PKM2 phosphorylation at Thr-328. **a** HA-tagged PKM2 was co-transfected with empty vector or Flag-tagged HSP90 in HEK293 cells. Representative IP experiments were performed to examine PKM2 Ser/Thr phosphorylation. Total cell lysates were subjected to immunoblotting analysis using specific antibodies against HA and Flag. Forced expression of Flag-tagged HSP90 in HEK293 cells increased the phosphorylation of HA-tagged PKM2. **b** Huh7 cells were transfected with empty vector or Flag-tagged HSP90. Representative IP experiments were performed to examine PKM2 Ser/Thr phosphorylation. Total cell lysates were subjected to immunoblotting analysis using specific antibodies against HSP90 and GAPDH. Overexpression of HSP90 increased PKM2 Ser/Thr phosphorylation in Huh7 cells. **c** Hep3B cells were transfected with negative control (NC) shRNA or HSP90 shRNA. Representative IP experiments were performed to examine PKM2 Ser/Thr phosphorylation. Total cell lysates were subjected to immunoblotting analysis using specific antibodies against HSP90 and GAPDH. Knockdown of HSP90 decreased PKM2 Ser/Thr phosphorylation in Hep3B cells. **d** Phosphor-specific protein enrichment and MS identified two putative phosphorylation sites in PKM2 regulated by HSP90. S405 and T326 residues were highlighted by red. **e** Huh7 cells were transfected with empty vector or Flag HSP90, along with PKM2-S405A mutant (Serine 405 to alanine mutation) or HA-T328A mutant (Threonine 328 to alanine mutation). T328A mutant, instead of S405A, abrogated the increased phosphorylation of PKM2 induced by HSP90 overexpression. **f** Huh7 cells were co-transfected with empty vector or Flag-HSP90, and, HA-tagged wild type (WT) PKM2 or HA-tagged T328A mutant PKM2. The protein half-life of HA-tagged WT or mutated PKM2 was analyzed following treatment with cycloheximide (CHX). HSP90 overexpression increased the half-life of the wild type PKM2 while failed to increase the half time of T328A PKM2 mutant

Post-translational modifications including phosphorylation [23–25] and acetylation [26] are critical for the protein stability and functional activity of PKM2. Previous study showed that PIM2 increased PKM2 protein levels by inducing the phosphorylation of PKM2 on the Thr-454 residue [25]. Therefore, we explored whether HSP90 increased PKM2 protein stability and abundance by modulating PKM2 phosphorylation. For this purpose, we co-transfected Flag-tagged HSP90 vector and HA-tagged PKM2 vector into HEK293T cells. Compared with the control vector, transfection of Flag-tagged HSP90 led to an increase in PKM2 phosphorylation on threonine/serine residues (Fig. 4a). Consistently, overexpression of HSP90 in Huh7 cells increased the phosphorylation of endogenous PKM2 protein on threonine/serine residues (Fig. 4b). On the other hand, knockdown of HSP90 decreased the threonine/serine phosphorylation of PKM2 in Hep3B cells (Fig. 4c and Additional File 5: Figure S4). Then, we performed the phosphor-specific protein enrichment and MS for 293 T cells overexpressing HSP90, to identify the exact phosphorylation site of PKM2 regulated by HSP90. The results of the phosphor-specific enrichment and MS demonstrated that Thr-328 and Ser-405 were two potential phosphorylation sites on PKM2 regulated by HSP90 (Fig. 4d). To determine which site was phosphorylated by HSP90, we generated phosphorylation mutants by mutating the threonine residue to alanine (S405A or T328A). Mutation of Ser-405 had no effect on the phosphorylation of PKM2 induced by HSP90-flag vector, while nutation of Thr-328 significantly blocked the phosphorylation of PKM2 induced by HSP90-flag vector (Fig. 4e). These data demonstrated that HSP90 led to the phosphorylation of PKM2 on Thr-328. Furthermore, we investigated whether HSP90 enhanced the protein stability of PKM2 through modulating phosphorylation of PKM2 on Thr-328. As shown in Fig. 4f, overexpression of HSP90 significantly enhanced the protein stability of wild type PKM2, but failed to enhance the stability of PKM2 mutant which was phosphorylation-defective at Thr-328. To further confirm that Thr-328 phosphorylation was critical for maintaining the stability and reducing the degradation of PKM2, we performed the ubiquitination assay for wild type PKM2 and T328A PKM2. As shown in Additional file 6: Figure S5, T328A PKM2 had significantly increased ubiquitination compared with the wild type PKM2 in HSP90 overexpressing Huh7 cells. To exclude the influence of endogenous PKM2 protein, we transfected Huh7 cells with PKM2 shRNA to knock down endogenous PKM2 protein (*P* < 0.05, Additional file 7: Figure S6A). Then, Huh7 cells with PKM2 knockdown were transfected with exogenous PKM2 vector (S405A or T328A) and HSP90 vector or control vector. Consistent with the data in Fig. 4e and f, mutation of Thr-328 blocked the increase of PKM2 phosphorylation (Additional file 7: Figure S6B) and stability (Additional file 7: Figure S6C) induced by HSP90 overexpression. These data demonstrated phosphorylation of PKM2 Thr-328 modulated by HSP90 was critical for maintaining the stability of PKM2 protein.

**Fig. 5:**
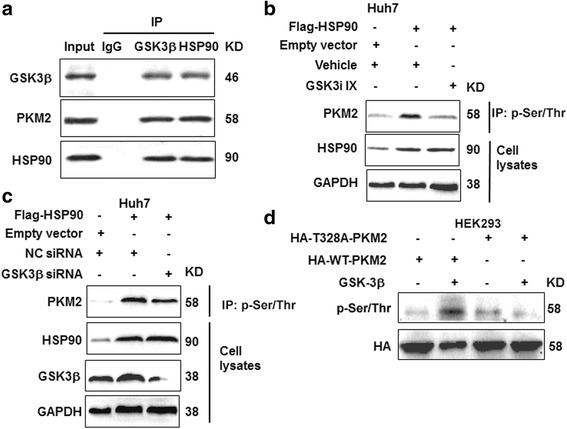
HSP90 increases PKM2 phosphorylation through GSK-3β. **a** Co-IP was performed for HSP90, PKM2 and GSK-3β to confirm whether these protein formed protein complex in Huh7 cells. **b** Huh7 cells transfected with HSP90 vector or control vector were treated with GSK3i IX, a GSK-3β inhibitor. Phosphorylation of PKM2 was examined by western blot after inhibiting GSK-3β activity. **c** Huh7 cells transfected with HSP90 vector or control vector were treated with GSK-3β siRNA to knockdown GSK-3β. Phosphorylation of PKM2 was examined by western blot after GSK-3β knockdown. **d** In vitro kinase assay to determine the effects of recombinant GSK3β on threonine phosphorylation of PKM2-WT or T328A

Since previous study demonstrated that HSP90 could interact with GSK-3β, which was a well-recognized protein kinase [27], we further explored if HSP90 regulated PKM2 phosphorylation through GSK3β. To answer this question, we first performed co-IP for HSP90, PKM2 and GSK-3β. We found that these three proteins indeed formed a protein complex together (Fig. 5a). More importantly, GSK-3β inhibitor (GSK3i IX) abrogated the increase of PKM2 phosphorylation induced by HSP90 overexpression (Fig. 5b). Knockdown of GSK-3β also blocked PKM2 phosphorylation induced by HSP90 overexpression (Fig 5c). Furthermore, in vitro kinase assay (Fig. 5d) showed that recombinant wild type GSK-3β increased the phosphorylation of wt-PKM2 while it had no effects on the T328A-PKM2, indicating GSK-3β could directly phosphorylate PKM2 at T328. Additionally, we investigated whether upstream pathway (PTEN/PI3K/Akt) of GSK3β affected PKM2 level through GSK3β. As shown in Additional file 8: Figure S7, knockdown of PTEN led to increased phosphorylation of Akt and resulted in increased level of PKM2. Knockdown of GSK3β partly abrogated the increase of PKM2 level induced by PTEN knockdown.

### Thr-328 phosphorylation is critical for the biological functions of PKM2 in HCC cells

**Fig. 6:**
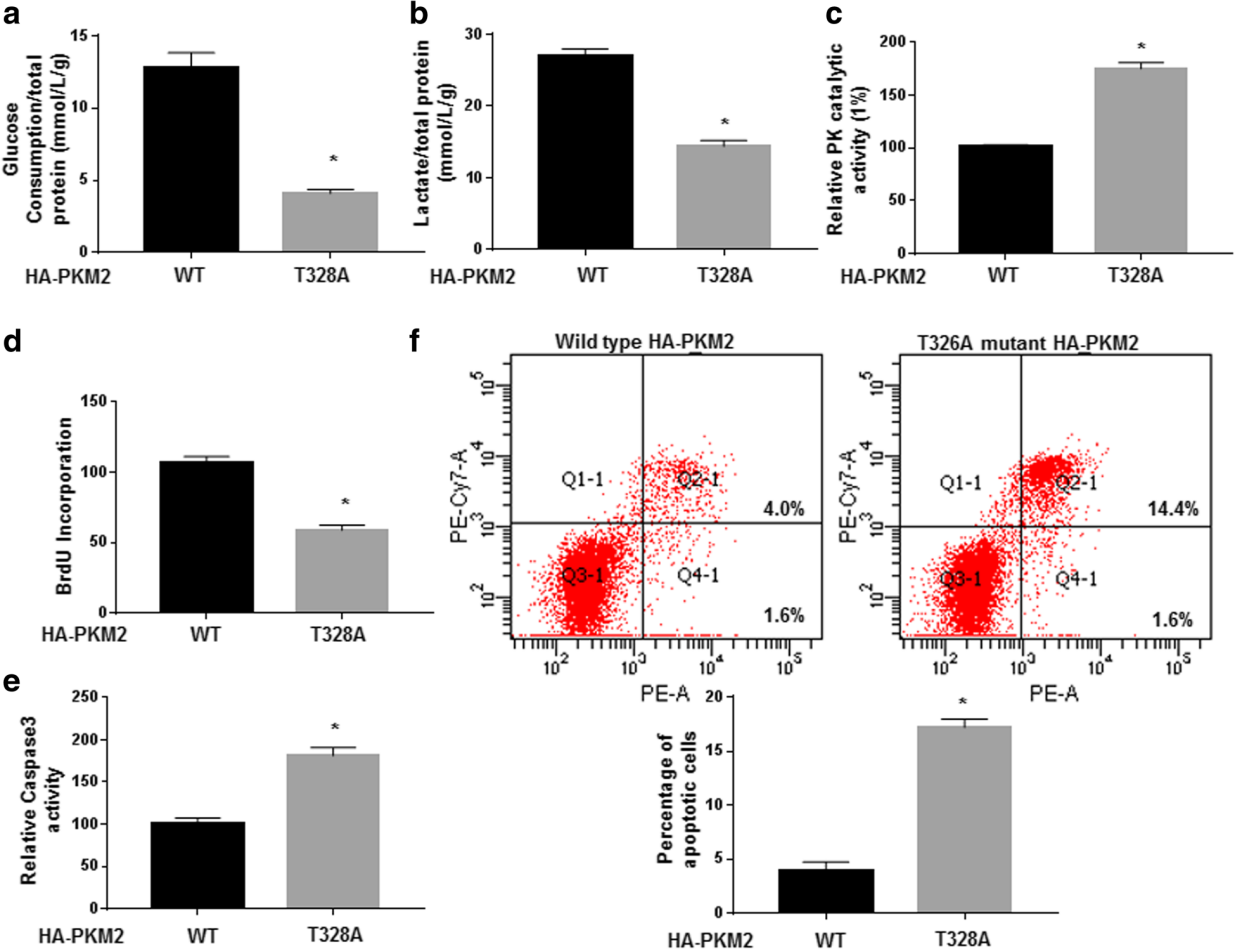
Thr-328 phosphorylation was required for the functional influence of PKM2 on glycolysis, proliferation and apoptosis of HCC cells. Compared with Huh7 cells transfected with wild-type PKM2, T328A PKM2 mutant significantly reduced (**a**) glucose consumption, (**b**) lactate production and (**c**) PK catalytic activity of Huh7 cells. Compared with Huh7 cells transfected with wild-type PKM2, Thr-328A mutated PKM2 significantly decreased the (**d**) proliferation and increased the (**e**) Caspase-3 activity and (**f**) apoptosis the of Huh7 cells. *, *P* < 0.05 by t test

PKM2 is a versatile protein implicated in glycolysis, mitochondrial function, cell proliferation and apoptosis. Additionally, PKM2 acts as a transcriptional cofactor to enhance HIF-1α and β-catenin mediated gene transcriptions [28, 29]. Therefore, we investigated whether Thr-328 phosphorylation of PKM2 was critical for its biological functions. Transfection of T328A PKM2 mutant into Huh7 cells significantly reduced glucose consumption and lactate production (*P* < 0.05, Fig. 6a and b). T328A mutant PKM2 in Huh7 cells resulted in a higher PK enzyme activity (*P* < 0.05, Fig. 6c). Furthermore, we investigated whether T328A mutant affected the cell proliferation and apoptosis. Compared with wild type PKM2, transfection of T328A mutant significantly decreased the proliferation and increased the Caspase-3 activity and apoptosis of Huh7 cells (*P* < 0.05, Fig. 6d-f). Similar effects of T328A mutant on glucose consumption, lactate production, PK enzyme activity, proliferation, Caspase-3 activity and apoptosis were observed in Huh7 cells with endogenous PKM2 knockdown (*P* < 0.05, Additional file 9: Figure S8A-8F). Additionally, T328A PKM2 mutant enhanced mitochondrial respiration suggested by increased oxygen consumption (*P* < 0.05, Additional file 10: Figure S9A and 9B). In contrast to wild type, T328A-mutant PKM2 significantly increased the release of 6-^14^CO_2_ from [6-^14^C] glucose while decreased the ratio of 1-^14^CO_2_ to 6-^14^CO_2_ (*P* < 0.05, Additional file 10: Figure S9C and 9D). These further supported that T328A PKM2 mutant enhanced mitochondrial respiration. Lastly, we examined whether T328A mutant affected the transcriptional cofactor functions of PKM2. In this regard, HEK293T cells were transfected with wild type PKM2 vector or T328A PKM2 mutant along with the p2.1 plasmid and pSV40-renilla. Then, cells were incubated in either normal cell incubator (20% O_2_) or hypoxic cell incubator (1% O_2_) for 24 h. As shown in Additional file 10: Figure S9E, T328A mutant significantly reduced the efficacy of PKM2 in activating the promoter under hypoxic condition (*P* < 0.05). T328A mutant also significantly reduced the efficacy of PKM2 in activating endogenous HIF-1α target genes and β-catenin target genes (*P* < 0.05, Additional file 10: Figure S9F and 9G). Taken together, these data indicated that Thr-328 phosphorylation was critical for PKM2 functions in regulating glycolysis, mitochondria respiration, cell proliferation, cell apoptosis and its cofactor functions.

### HSP90 promotes glycolysis and proliferation and reduces apoptosis of HCC cells in a PKM2 dependent manner

**Fig. 7:**
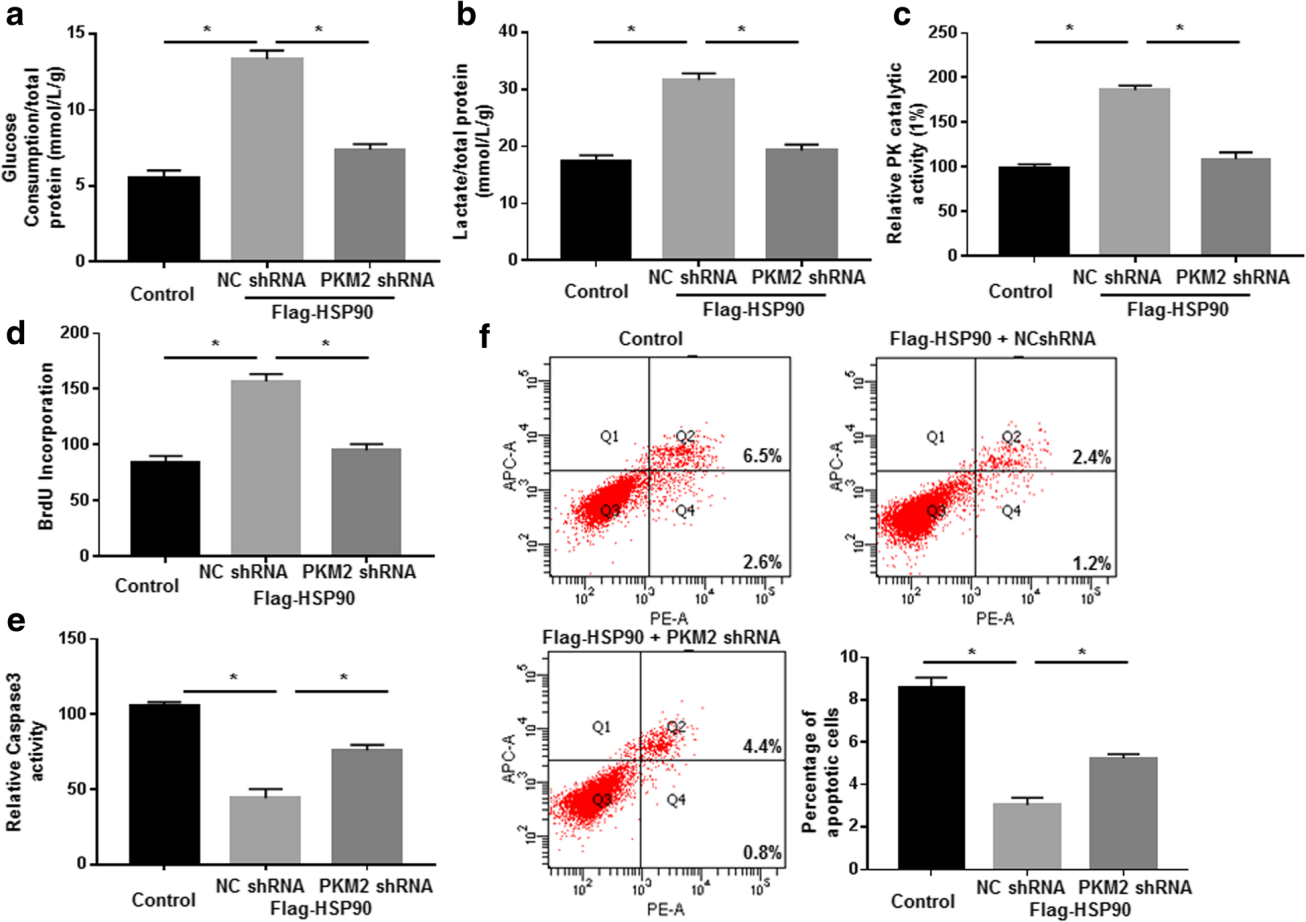
HSP90 enhanced the glycolysis and proliferation while decreased the apoptosis of HCC cells through PKM2. Huh7 cells were co-transfected with corresponding vectors. Overexpression of HSP90 significantly increased (**a**) glucose consumption, (**b**) lactate production and (**c**) PK activity of Huh7 cells. PKM2 knockdown abrogated the promoting effects of HSP90 on glycolysis. Furthermore, overexpression of HSP90 significantly (**d**) increased proliferation while decreased the (**e**) Caspase-3 activity and (**f**) apoptosis of Huh7 cells. PKM2 knockdown abrogated the regulatory effects of HSP90 overexpression on cell proliferation and apoptosis. *, P < 0.05 by t test

Then, we investigated whether HSP90 regulated the glycolysis, proliferation and apoptosis of cancer cells through PKM2. Huh7 cells that were transfected with HSP90 vector showed significantly increased glucose consumption, lactate production and PK activity (*P* < 0.05, Fig. 7a-c), and these promoting effects on cell glycolysis was abrogated by PKM2 knockdown (*P* < 0.05, Fig. 7a-c). Additionally, we also found that HSP90 overexpression significantly enhanced cell proliferation and inhibited Caspase-3 activity and apoptosis in a PKM2 dependent manner (*P* < 0.05, Fig. 7d-f). These regulatory effects of HSP90 were reversed by PKM2 knockdown (*P* < 0.05, Fig. 7d-f). These data demonstrate that HSP90 regulates the glycolysis, proliferation and apoptosis of HCC cells in a PKM2 dependent manner.

### HSP90 promotes the growth of HCC cells by regulating PKM2 in vivo

**Fig. 8:**
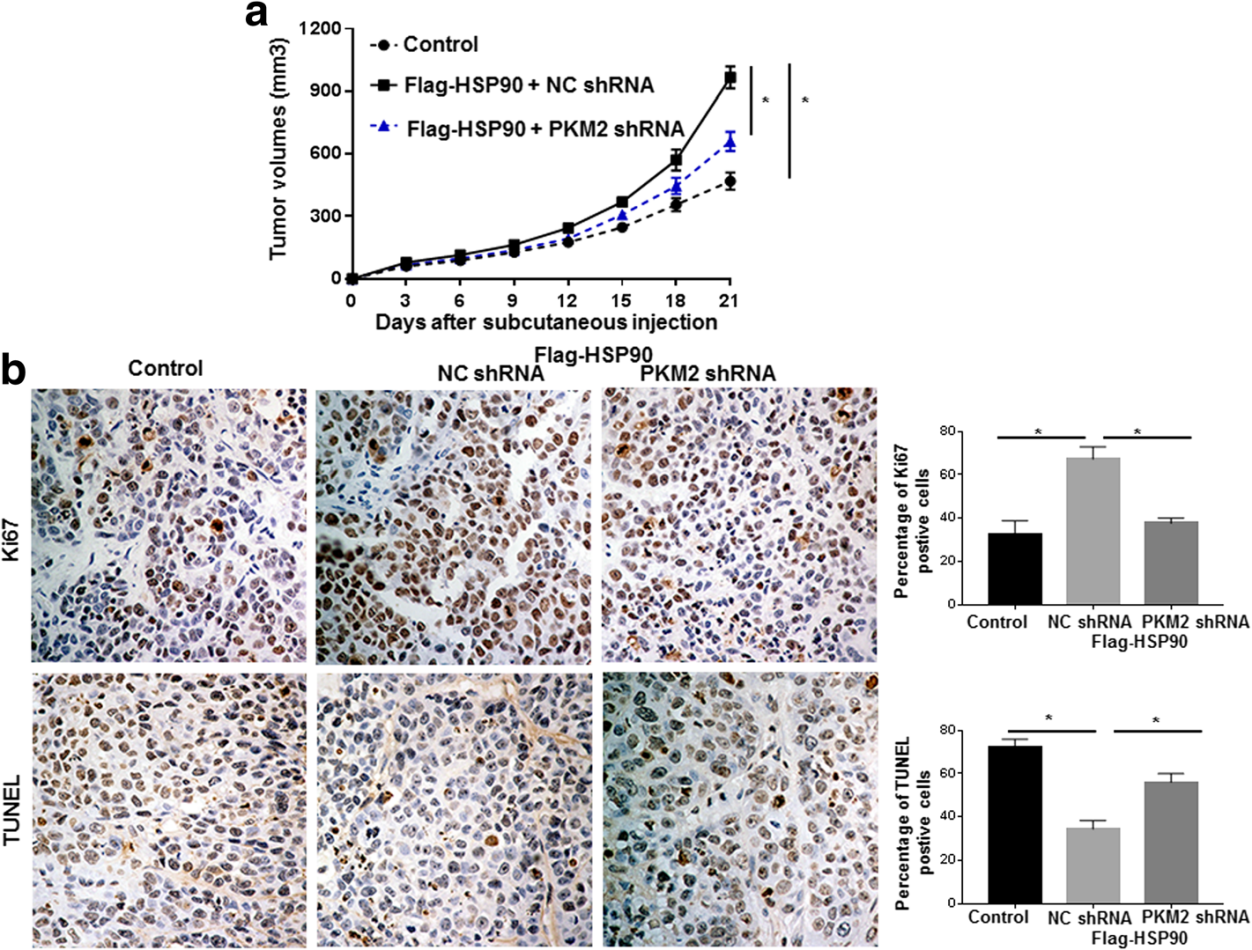
HSP90 promoted the growth of Huh7 cells through PKM2 in vivo. **a** Subcutaneous injection was performed using Huh7 cells co-transfected with corresponding vectors. Overexpression of HSP90 significantly increased the growth of Huh7 cells in nude mice while PKM2 knockdown abrogated the promoting effects of HSP90 on the growth of Huh7 cells in vivo. *, *P* < 0.05 by two-way ANOVA. **b** Tumor nodules were subjected to IHC staining for Ki-67, TUNEL assays and quantitative analysis. IHC staining for Ki67 and TUNEL assays revealed that HSP90 overexpression significantly increased the number of Ki-67 positive cells and decreased the number of apoptotic cells. These effects induced by HSP90 overexpression were abrogated by PKM2 knockdown. *, *P* < 0.05 by One-way ANOVA

In vivo experiments were performed to further confirm our findings in vitro. As shown in Fig. 8a, overexpression of HSP90 significantly increased the growth of Huh7 cells in nude mice (*P* < 0.05). The promoting effects of HSP90 on the growth of Huh7 cells were blocked by PKM2 knockdown in vivo (*P* < 0.05, Fig. 8a). Ki67 staining and TUNEL staining further confirmed that HSP90 overexpression enhanced the proliferation and decreased apoptosis of Huh7 cells in nude mice (*P* < 0.05, Fig. 8b), and these regulatory effects of HSP90 on the proliferation and apoptosis of Huh7 cells were abrogated by PKM2 knockdown in vivo (*P* < 0.05, Fig. 8b). These data demonstrate that HSP90 promotes the growth of HCC cells through PKM2 in vivo.

### Overexpression of HSP90 and PKM2 predicts the poor prognosis of HCC patients

**Fig. 9:**
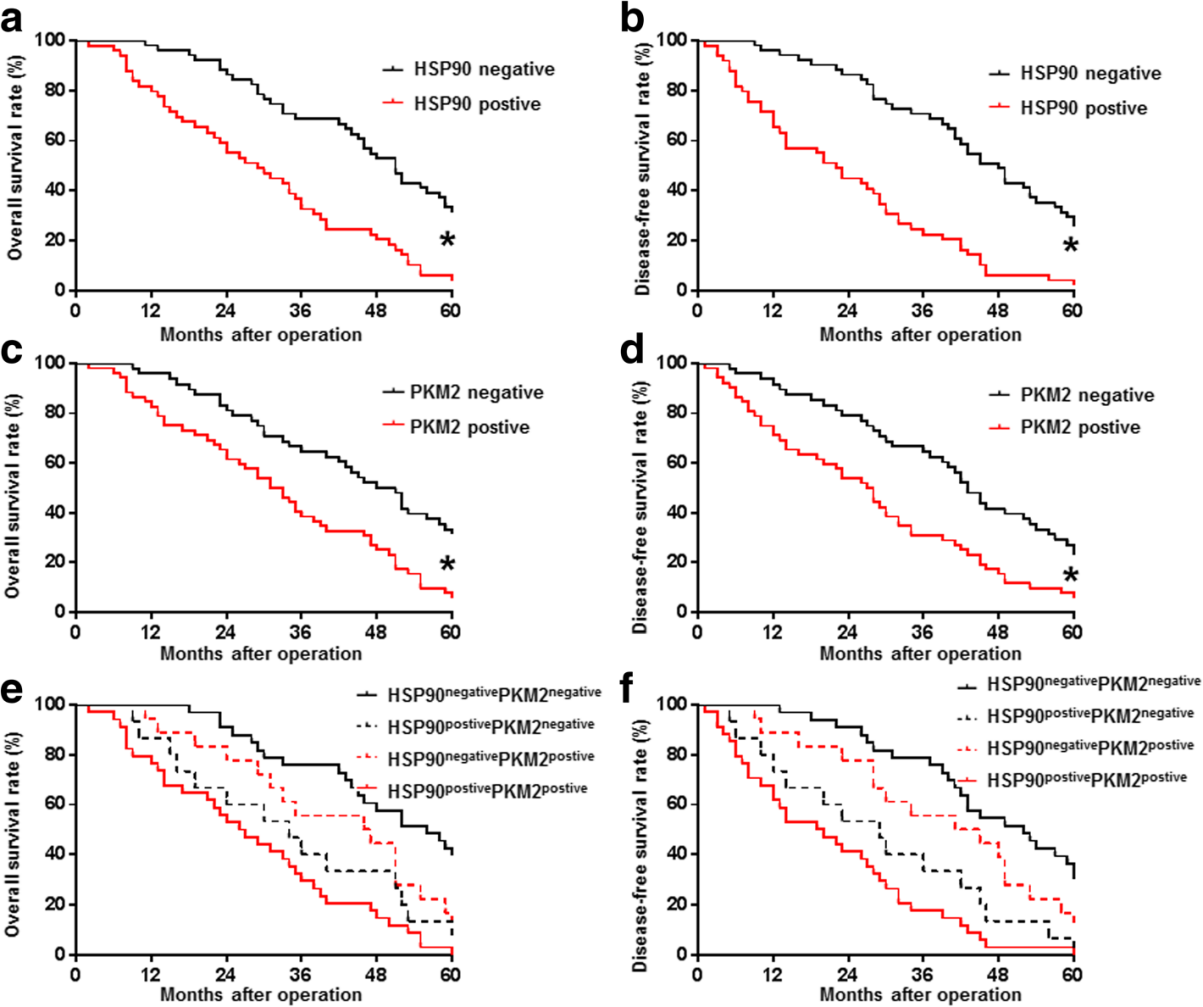
The prognostic value of HSP90 and PKM2 for HCC patients. HCC patients were divided into HSP90 positive group and HSP90 negative group based on IHC staining. Patients with positive expression of HSP90 had significant shorter (**a**) overall survival (OS) and (**b**) disease free survival (DFS). HCC patients were divided into PKM2 positive group and PKM2 negative group based on IHC staining. Patients with positive expression of PKM2 had obvious poorer (**c**) OS and (**d**) DFS. HCC patients were divided into four groups: HSP90^postive^PKM2^postive^ group, HSP90^postive^ PKM2^negative^ group, HSP90^negative^PKM2^postive^ group and HSP90^negative^PKM2^negative^ group. HCC patients in HSP90^negative^PKM2^negative^ group had the best (**e**) OS and (**f**) DFS while those in HSP90^postive^PKM2^postive^ group had the lowest (**e**) OS and (**f**) DFS. *, *P* < 0.05 by Log-rank test

Next, we examined the prognostic value of HSP90 and PKM2 in HCC. As shown in Table 1, expression of HSP90 was correlated with unfavorable clinicopathological features of HCC patients including large tumor size (*P* = 0.006), portal vein tumor thrombus (PVTT) (*P* = 0.002) and advanced tumor-node-metastasis (TNM) stage (*P* = 0.005). PKM2 positive expression was associated with high alpha fetoprotein (AFP) level (*P* = 0.015), large tumor size (*P* = 0.003), occurrence of PVTT (*P* = 0.002) and advanced TNM stage (*P* = 0.008). More importantly, HCC patients with positive HSP90 expression had significant shorter overall survival (OS) (*P* < 0.001, Fig. 9a) and disease-free survival (DFS) (*P* < 0.001, Fig. 9b). In accordance, positive PKM2 expression was also correlated with reduced OS (*P* = 0.0035, Fig. 9c) and DFS (*P* = 0.003, Fig. 9d). Finally, we divided HCC patients into four subgroups based on the expression status of HSP90 and PKM2. HCC Patients with positive expression of both HSP90 and PKM2 had the worst OS and DFS while those with negative expression of HSP90 and PKM2 had the best OS and DFS (Fig. 9e and f). Thus, HSP90, PKM2 and the combination of these two proteins predict the prognosis of HCC patients.

## Discussion

Accumulating evidence demonstrates that the molecular chaperone HSP90 plays critical roles in modulating the conformation, stability and function of oncogenic proteins, which were involved in cell proliferation, apoptosis, cell cycle progression, migration, invasion and drug resistance [16, 17, 30, 31]. Previous studies demonstrated that HSP90 was overexpressed in HCC tissues and promoted the growth of HCC cells. However, the detailed mechanisms by which HSP90 promotes the progression of HCC remain largely unknown. In this study, tandem affinity purification recognized HSP90 as a novel binding partner for PKM2. IP assay and GST pull-down assay further confirmed that HSP90 could directly bind to PKM2 protein. Furthermore, we found that overexpression of HSP90 increased the expression of PKM2 in Huh7 cells while HSP90 knockdown reduced PKM2 level in Hep3B cells. The regulatory effect of HSP90 on PKM2 level was further confirmed by the IHC staining results in HCC tissues. IHC staining data showed that the expression level of HSP90 was positively correlated with PKM2 level in HCC tissues.

To further determine the mechanisms by which HSP90 increases PKM2 level in HCC cells, we first performed qRT-PCR to confirm whether HSP90 could promote the transcription of PKM2. However, neither HSP90 overexpression nor HSP90 knockdown affected the mRNA level of PKM2. This indicated that HSP90 increased PKM2 level through a post-transcriptional mechanism. Previous study demonstrated that HSP90 was an important regulator of the folding, transportation and stability of various proteins in cells [16]. Therefore, we investigated whether HSP90 could affect the protein stability of PKM2. The data of western blot showed that overexpression of HSP90 significantly increased the half-time of PKM2 in Huh7 while HSP90 knockdown decreased the half-time of PKM2 in Hep3B cells. Furthermore, blocking the proteasomal degradation with MG-132 reversed the downregulation of PKM2 induced by HSP90 knockdown. These data demonstrate that HSP90 is critical for reducing the proteasomal degradation of PKM2 and thus increasing the stability of PKM2. Interestingly, another study in lung cancer also showed that PKM2 could directly interacted with heat-shock protein 90 (HSP90), and thus increase the stability EGFR. This means the interaction between PKM2 and HSP90 may be a common phenomenon in difference types of human cancer, and, is critical for the progression of human cancers through many different mechanisms.

PKM2 was a well-known regulator of the cancer cell metabolism, proliferation, apoptosis and metastasis, and was found to be significantly increased in many types of human cancers [32, 33]. Accumulating studies found that post-translational modifications and protein-protein interaction were critical for regulating the enzymatic activity and protein stability of PKM2 [23–25]. Previous study showed that PIM2 could bind to PKM2 and led to the phosphorylation of PKM2 at Thr-454. Thr-454 phosphorylation inhibited the enzymatic activity of PKM2 and was critical for the protein stability of PKM2 [33]. In this study, we demonstrated that overexpression of HSP90 led to increased phosphorylation of PKM2 while HSP90 knockdown reduced PKM2 phosphorylation. Phospho-specific enrichment technology and site-mutation of PKM2 protein further confirmed that HSP90 led to the phosphorylation of PKM2 at Thr-328. Moreover, we found Thr-328 phosphorylation was critical for maintaining the protein stability of PKM2. Functionally, we demonstrated that phosphorylation of PKM2 at Thr-328 was critical for its biological functions including regulating glycolysis, mitochondria respiration, cell apoptosis, proliferation and co-factor functions. However, different from PIM2, HSP90 protein was not a serine/threonine kinase. Previous study showed that GSK-3β, a canonical protein kinase [27], could bind to HSP90. Therefore, we hypothesized that GSK-3β participated in HSP90-mediated PKM2 phosphorylation. Our data showed GSK-3β, HSP90 and PKM2 indeed formed a protein complex together. Inhibiting GSK-3β activity or expression abrogated the increase of PKM2 phosphorylation induced by HSP90 overexpression. Taken together, we concluded that HSP90 led to PKM2 phosphorylation at Thr-328 induced by HSP90 was dependent on GSK-3β.

After confirming that HSP90 could bind with PKM2 and regulate the phosphorylation and protein stability of PKM2, we further investigated whether HSP90 could affect the glycolysis, proliferation and apoptosis of HCC cells through PKM2. Functional assay showed that overexpression of HSP90 enhanced the glycolysis and proliferation, and reduced the apoptosis of HCC cells. Knockdown of PKM2 abrogated these functional influences of HSP90 on HCC cells. In vivo experiments further confirmed that overexpression of HSP90 potentiated the growth of HCC cells and PKM2 knockdown blocked the promoting effect of HSP90 on HCC growth. Therefore, these data demonstrate that HSP90 not only binds to PKM2 and regulates its phosphorylation but also exerts its oncogenic functions through PKM2 in HCC.

In all, this study identified for the first time that HSP90 was a novel binding partner with PKM2 in HCC cells and enhanced the protein abundance of PKM2 in HCC. We also demonstrated that the interaction between HSP90 and PKM2 led to phosphorylation of PKM2 at Thr-328. GSK-3β, formed a protein complex with HSP90 and PKM2, and mediated Thr-328 phosphorylation of PKM2 induced by HSP90. Thr-328 phosphorylation of PKM2, a novel identified phosphorylation site, was critical for its functions in regulating glycolysis, mitochondria respiration, cell proliferation and apoptosis. Functionally, we demonstrated that HSP90 could potentiate the glycolysis and proliferation, reduce the apoptosis and thus enhanced the growth of HCC cells through PKM2.

## Conclusions

To conclude, we recognize HSP90 as a novel binding partner of PKM2 in HCC cells. HSP90 regulates PKM2 abundance by enhancing the protein stability and reducing the proteasomal degradation. PKM2 Thr-328 phosphorylation, which is mediated by HSP90/GSK-3β complex, is critical for the protein stability and biological functions of PKM2 in HCC. Moreover, HSP90 promoted glycolysis and proliferation, and inhibited apoptosis of HCC cells through PKM2. Notably, overexpression of HSP90 and PKM2 significantly predict poor prognosis of HCC patients. Our findings highlight a novel mechanism of HSP90 in the progression of HCC and provide HSP90/PKM2 axis as a promising drug target for HCC.

## Additional files

Additional file 1: Table S1. The primer sequences used in this study (DOCX 16 kb)

Additional file 2: Figure S1. Knockdown of HSP90 reduced the protein level of PKM2. Hep3B cells were transfected with HSP90 shRNA or negative-control (NC) shRNA. 72 h after transfection, the levels of HSP90 and PKM2 protein in Hep3B cells were examined. Knockdown of HSP90 decreased PKM2 protein in Hep3B cells. (TIFF 4220 kb)

Additional file 3: Figure S2. HSP90 inhibitors reduced the protein level of PKM2. Hep3B cells were treated with 17-AAG and 17-DMAG, two kinds of HSP90 inhibitors. HSP90 inhibitors decreased PKM2 protein in Hep3B cells. (TIFF 2886 kb)

Additional file 4: Figure S3. MG132 restored the protein level of PKM2 induced by HSP90 knockdown. MG-132 was used to inhibit the proteasomal degradation in Hep3B cells. MG-132 treatment led to PKM2 accumulation in the detergent insoluble fraction of the cell lysate. (TIFF 7067 kb)

Additional file 5: Figure S4. HSP90 knockdown led to decreased Ser/Thr phosphorylation of PKM2. Hep3B cells were transfected with negative control (NC) shRNA or HSP90 shRNA. Representative IP experiments were performed to examine PKM2 Ser/Thr phosphorylation. Total cell lysates were subjected to immunoblotting analysis using specific antibodies against HSP90 and GAPDH. (TIFF 4128 kb)

Additional file 6: Figure S5. Thr-328 phosphorylation induced by HSP90 decreased the ubiquitination of PKM2 protein. Huh7 cell that were transfected with Flag-HSP90 were then transfected with HA-tagged wild type PKM2 or HA-tagged T328A PKM2. Proteins pull-down by HA antibody was subjected to western blot for ubiquitination. (TIFF 258 kb)

Additional file 7: Figure S6. Thr-328 phosphorylation-induced by HSP90 overexpression was critical for maintaining the stability of PKM2. A) PKM2 shRNA was used to knock down exogenous PKM2 in Huh7 cells. PKM2 shRNA effectively depleted the expression of endogenous PKM2 protein. B) Huh7 cells with endogenous PKM2 depleted were transfected with corresponding vectors. T328A mutant, instead of S405A, abrogated the increased phosphorylation of PKM2 induced by HSP90 overexpression. C) Huh7 cells with endogenous PKM2 depleted were co-transfected with corresponding vectors. The protein half-life of HA-tagged WT or mutated PKM2 was analyzed following treatment with cycloheximide (CHX). HSP90 overexpression increased the half-life of the wild type PKM2 while failed to increase the half time of T328A PKM2 mutant. *, *P* < 0.05 by t test. (TIFF 1330 kb)

Additional file 8: Figure S7. Knockdown of GSK-3β partly inhibited the elevation of PKM2 protein induced by PTEN knockdown. Huh7 cells transfected with PTEN siRNA or control siRNA along with or without GSK-3β. PTEN knockdown, which activated PT3K/AKT pathway, led to increased level of PKM2. Knockdown of GSK-3β partly inhibited the elevation of PKM2 protein induced by PTEN knockdown. (TIFF 6810 kb)

Additional file 9: Figure S8. Thr-328 phosphorylation-induced by HSP90 overexpression was critical for maintaining the function of PKM2. In Huh7 cells with endogenous PKM2 depleted, transfection of T328A PKM2 mutant significantly reduced (A) glucose consumption, (B) lactate production and (C) PK catalytic activity of Huh7 cells. Furthermore, transfection of T328A PKM2 mutant significantly reduced (D) proliferation and increased the (E) Caspase-3 activity and (F) apoptosis the of Huh7 cells. (TIFF 1030 kb)

Additional file 10: Figure S9. Thr-328 phosphorylation was required for PKM2 to regulate mitochondria respiration and co-factor function. Huh7 cells were transfected with HA-tagged PKM2-WT or T454A. Cells were re-plated into appropriate plates for analysis of O_2_ consumption, OCR, 6-^14^CO_2_ and ratio of 1-^14^CO_2_ to 6-^14^CO_2_. Compared with Huh7 cells transfected with wild-type PKM2, T328A PKM2 mutant significantly increased (A) O_2_ consumption, (B) OCR, (C) 6-^14^CO_2_ release from [6-^14^C] glucose while decreased (D) ratio of 1-^14^CO_2_ to 6-^14^CO_2_. E) HEK293T cells were co-transfected with wild type PKM2 or T328A PKM2 mutant, p2.1 and pSV40-Renilla. Transfected cells were exposed to 20% O_2_ or 1% O_2_ for 24 h. The ratio of firefly to renilla luciferase activity was determined. T328A PKM2 mutant significantly reduced the ability of PKM2 to promote hypoxia-induced gene transcription. Huh7 cells were transfected with wild type PKM2 or T328A PKM2 mutant. Two days after transfection, the cells were exposed to 1% O_2_ or 20% O_2_ for another 24 h. The mRNA levels of indicated genes were examined by qRT-PCR. T328A pKM2 mutant significantly reduced the ability of PKM2 in activating endogenous (F) HIF-1α target genes and (G) β-catenin target genes. *, *P* < 0.05 by t test. (TIFF 270 kb)

## Abbreviations

AFP: Alpha fetoprotein
DMEM: Dulbecco’s modified Eagle’s medium
FBS: Fetal bovine serum
GSK-3β: Glycogen synthase kinase-3β
GST: Glutathione S transferase
HCC: Hepatocellular carcinoma
HSP90: Heat shock protein 90
IHC: Immunohistochemistry
IP: Immunoprecipitation
MS: Mass spectrometry
PKM2: Pyruvate kinase M2
PVTT: Portal vein tumor thrombus
qRT-PCR: Real-time quantitative reverse transcription polymerase chain reaction
SDS-PAGE: Sodium dodecyl sulphate-polyacrylamide gel electrophoresis
TNM: Tumor-node-metastasis

## References

[1] Venook AP, Papandreou C, Furuse J, de Guevara LL (2010). The incidence and epidemiology of hepatocellular carcinoma: a global and regional perspective. Oncologist.

[2] Ferenci P, Fried M, Labrecque D, Bruix J, Sherman M, Omata M, Heathcote J, Piratsivuth T, Kew M, Otegbayo JA (2010). Hepatocellular carcinoma (HCC): a global perspective. J Clin Gastroenterol.

[3] Ryder SD (2003). Guidelines for the diagnosis and treatment of hepatocellular carcinoma (HCC) in adults. Gut.

[4] Mazurek S, Boschek CB, Hugo F, Eigenbrodt E. Pyruvate kinase type M2 and its role in tumor growth and spreading. Semin Cancer Biol. 2005;15(4):300–8. 10.1016/j.semcancer.2005.04.009 15908230

[5] Christofk HR, Vander Heiden MG, Wu N, Asara JM, Cantley LC (2008). Pyruvate kinase M2 is a phosphotyrosine-binding protein. Nature.

[6] Mazurek S (2011). Pyruvate kinase type M2: a key regulator of the metabolic budget system in tumor cells. Int J Biochem Cell Biol.

[7] Mazurek S, Grimm H, Boschek C, Vaupel P, Eigenbrodt E (2002). Pyruvate kinase type M2: a crossroad in the tumor metabolome. Br J Nutr.

[8] Luo W, Semenza GL (2011). Pyruvate kinase M2 regulates glucose metabolism by functioning as a coactivator for hypoxia-inducible factor 1 in cancer cells. Oncotarget.

[9] Ferguson EC, Rathmell JC (2008). New roles for pyruvate kinase M2: working out the Warburg effect. Trends Biochem Sci.

[10] Shang Y, He J, Wang Y, Feng Q, Zhang Y, Guo J, Li J, Li S, Wang Y, Yan G (2017). CHIP/Stub1 regulates the Warburg effect by promoting degradation of PKM2 in ovarian carcinoma. Oncogene.

[11] Liu K, Li F, Han H, Chen Y, Mao Z, Luo J, Zhao Y, Zheng B, Gu W, Zhao W (2016). Parkin regulates the activity of Pyruvate Kinase M2. J Biol Chem.

[12] Kim SR, Kim JO, Lim KH, Yun JH, Han I, Baek KH (2015). Regulation of pyruvate kinase isozyme M2 is mediated by the ubiquitin-specific protease 20. Int J Oncol.

[13] Xu Q, Liu X, Zheng X, Yao Y, Liu Q (2014). PKM2 regulates Gli1 expression in hepatocellular carcinoma. Oncol Lett.

[14] Sarto C, Binz PA, Mocarelli P (2000). Heat shock proteins in human cancer. Electrophoresis.

[15] Trepel J, Mollapour M, Giaccone G, Neckers L (2010). Targeting the dynamic HSP90 complex in cancer. Nat Rev Cancer.

[16] Taipale M, Jarosz DF, Lindquist S (2010). HSP90 at the hub of protein homeostasis: emerging mechanistic insights. Nat Rev Mol Cell Biol.

[17] Liu X, Chen S, Tu J, Cai W, Xu Q (2016). HSP90 inhibits apoptosis and promotes growth by regulating HIF-1α abundance in hepatocellular carcinoma. Int J Mol Med.

[18] Tu K, Zheng X, Zan X, Han S, Yao Y, Liu Q (2012). Evaluation of Fbxw7 expression and its correlation with the expression of c-Myc, cyclin E and p53 in human hepatocellular carcinoma. Hepatol Res.

[19] Dou C, Wang Y, Li C, Liu Z, Jia Y, Li Q, Yang W, Yao Y, Liu Q, Tu K (2015). MicroRNA-212 suppresses tumor growth of human hepatocellular carcinoma by targeting FOXA1. Oncotarget.

[20] Xu Q, Yang C, Du Y, Chen Y, Liu H, Deng M, Zhang H, Zhang L, Liu T, Liu Q, Wang L, Lou Z, Pei H. AMPK regulates histone H2B O-GlcNAcylation. Nucleic Acids Res. 2014;42(9):5594–604. 10.1093/nar/gku236 PMC4027166 24692660

[21] Tu K, Yang W, Li C, Zheng X, Lu Z, Guo C, Yao Y, Liu Q (2014). Fbxw7 is an independent prognostic marker and induces apoptosis and growth arrest by regulating YAP abundance in hepatocellular carcinoma. Mol Cancer.

[22] Dou C, Liu Z, Xu M, Jia Y, Wang Y, Li Q, Yang W, Zheng X, Tu K, Liu Q (2016). miR-187-3p inhibits the metastasis and epithelial–mesenchymal transition of hepatocellular carcinoma by targeting S100A4. Cancer Lett.

[23] Yang W, Zheng Y, Xia Y, Ji H, Chen X, Guo F, Lyssiotis CA, Aldape K, Cantley LC, Lu Z (2012). ERK1/2-dependent phosphorylation and nuclear translocation of PKM2 promotes the Warburg effect. Nat Cell Biol.

[24] Hitosugi T, Kang S, Vander Heiden MG, Chung T-W, Elf S, Lythgoe K, Dong S, Lonial S, Wang X, Chen GZ (2009). Tyrosine phosphorylation inhibits PKM2 to promote the Warburg effect and tumor growth. Sci Signal.

[25] Yu Z, Zhao X, Huang L, Zhang T, Yang F, Xie L, Song S, Miao P, Zhao L, Sun X (2013). Proviral insertion in murine lymphomas 2 (PIM2) oncogene phosphorylates pyruvate kinase M2 (PKM2) and promotes glycolysis in cancer cells. J Biol Chem.

[26] Lv L, Li D, Zhao D, Lin R, Chu Y, Zhang H, Zha Z, Liu Y, Li Z, Xu Y (2011). Acetylation targets the M2 isoform of pyruvate kinase for degradation through chaperone-mediated autophagy and promotes tumor growth. Mol Cell.

[27] Banz VM, Medova M, Keogh A, Furer C, Zimmer Y, Candinas D, Stroka D (2009). Hsp90 transcriptionally and post-translationally regulates the expression of NDRG1 and maintains the stability of its modifying kinase GSK3beta. Biochim Biophys Acta.

[28] Luo W, Hu H, Chang R, Zhong J, Knabel M, O'Meally R, Cole RN, Pandey A, Semenza GL (2011). Pyruvate kinase M2 is a PHD3-stimulated coactivator for hypoxia-inducible factor 1. Cell.

[29] Yang W, Xia Y, Ji H, Zheng Y, Liang J, Huang W, Gao X, Aldape K, Lu Z (2011). Nuclear PKM2 regulates beta-catenin transactivation upon EGFR activation. Nature.

[30] Tsutsumi S, Neckers L (2007). Extracellular heat shock protein 90: a role for a molecular chaperone in cell motility and cancer metastasis. Cancer Sci.

[31] Jhaveri K, Modi S (2011). HSP90 inhibitors for cancer therapy and overcoming drug resistance. Advances in pharmacology (San Diego, Calif).

[32] Wong N, De Melo J, Tang D (2013). PKM2, a central point of regulation in cancer metabolism. Int J Cell Biol.

[33] Luo W, Semenza GL (2012). Emerging roles of PKM2 in cell metabolism and cancer progression. Trends Endocrinol Metab.

