# Supplementary material for: HSP90 promotes cell glycolysis, proliferation and inhibits apoptosis by regulating PKM2 abundance via Thr-328 phosphorylation in hepatocellular carcinoma
Source: Mol Cancer. 2017 Dec 20;16:178. doi: 10.1186/s12943-017-0748-y (PMC5738801; doi:10.1186/s12943-017-0748-y)
Supplement: Supplementary file 1 — The primer sequences used in this study (DOCX 16 kb) [file 12943_2017_748_MOESM1_ESM.docx]

**Additional file 1: Table S1. The primer sequences used in this study**

| PKM2 | Forward | 5'-ACTCGGGCTGAAGGCAGTGA-3’ |
| --- | --- | --- |
|  | Reverse | 5'-TGTGGGGTCGCTGGTAATGG-3’ |
| Glut1 | Forward | 5'-CAGTTCGGCTATAACACTGGTG-3’ |
|  | Reverse | 5'-GCCCCCGACAGAGAAGATG-3’ |
| ENO1 | Forward | 5’-TGCCGTCTGCAAAGCTGGTG-3’ |
|  | Reverse | 5’-CGCATGGCTTCCCTGAAGTT-3’ |
| VEGF | Forward | 5'-CTTGCCTTGCTGCTCTAC-3’ |
|  | Reverse | 5'-TGGCTTGAAGATGTACTCG-3’ |
| LDHA | Forward | 5'-ATCTTGACCTACGTGGCTTGGA-3’ |
|  | Reverse | 5'-CCATACAGGCACACTGGAATCTC-3’ |
| PDK1 | Forward | 5'-CTTCAGCCGCAGCTTCAGCT-3’ |
|  | Reverse | 5'-CAACTCTTGCCGCAGAAACA-3’ |
| CCND1 | Forward | 5'-TGCCAGGAGCAGATCGAAGC-3’ |
|  | Reverse | 5'-TCAGATGTCCACGTCCCGCA-3’ |
| Myc | Forward | 5’-GAAGGGCAGGGCTTCTCAGAGGCTT-3’ |
|  | Reverse | 5’-TATTCGCTCCGGATCTCCCTTCCC-3’ |
| GAPDH | Forward | 5’-AGGGCTGCTTTTAACTCTGGT-3’ |
|  | Reverse | 5’-CCCCACTTGATTTTGGAGGGA-3’ |
