# Supplementary figures and images for: HSP90 promotes cell glycolysis, proliferation and inhibits apoptosis by regulating PKM2 abundance via Thr-328 phosphorylation in hepatocellular carcinoma
Source: Mol Cancer. 2017 Dec 20;16:178. doi: 10.1186/s12943-017-0748-y (PMC5738801; doi:10.1186/s12943-017-0748-y)

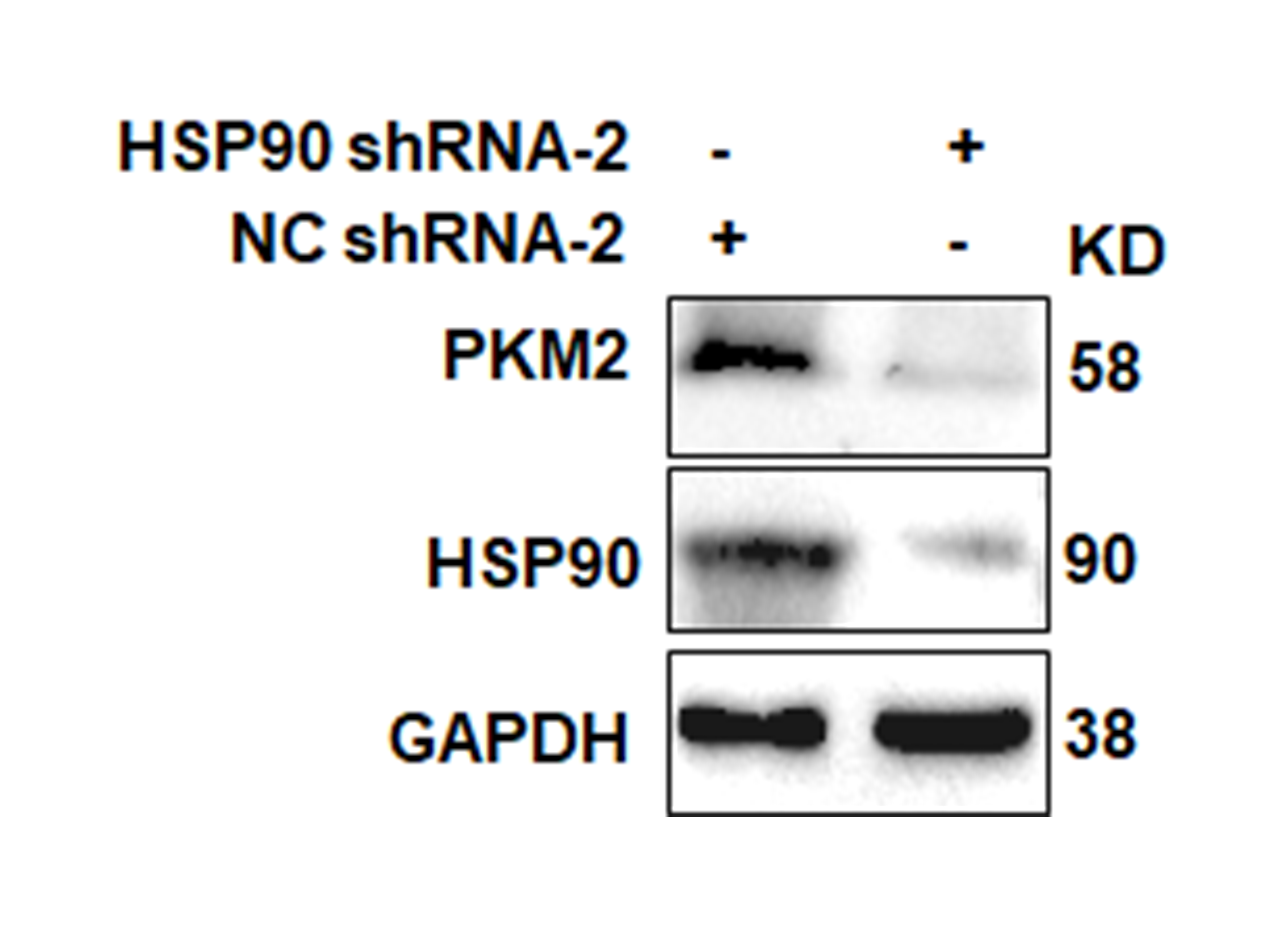

Supplement: Supplementary file 2 — Knockdown of HSP90 reduced the protein level of PKM2. Hep3B cells were transfected with HSP90 shRNA or negative-control (NC) shRNA. 72 h after transfection, the levels of HSP90 and PKM2 protein in Hep3B cells were examined. Knockdown of HSP90 decreased PKM2 protein in Hep3B cells. (TIFF 4220 kb) [file 12943_2017_748_MOESM2_ESM.tif]

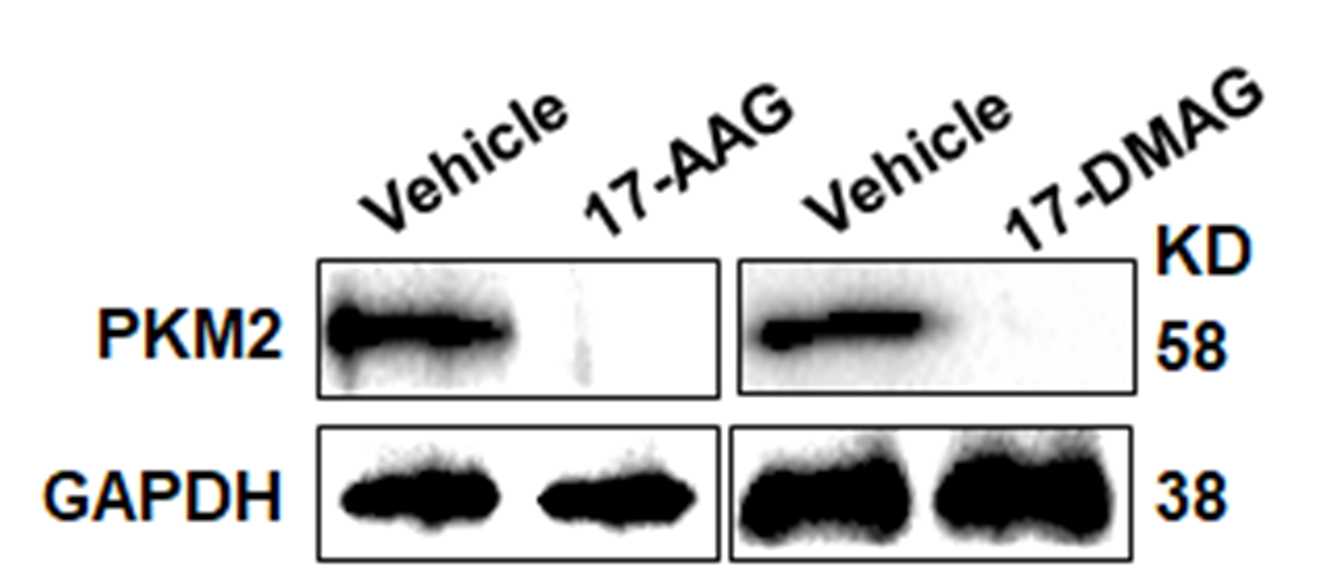

Supplement: Supplementary file 3 — HSP90 inhibitors reduced the protein level of PKM2. Hep3B cells were treated with 17-AAG and 17-DMAG, two kinds of HSP90 inhibitors. HSP90 inhibitors decreased PKM2 protein in Hep3B cells. (TIFF 2886 kb) [file 12943_2017_748_MOESM3_ESM.tif]

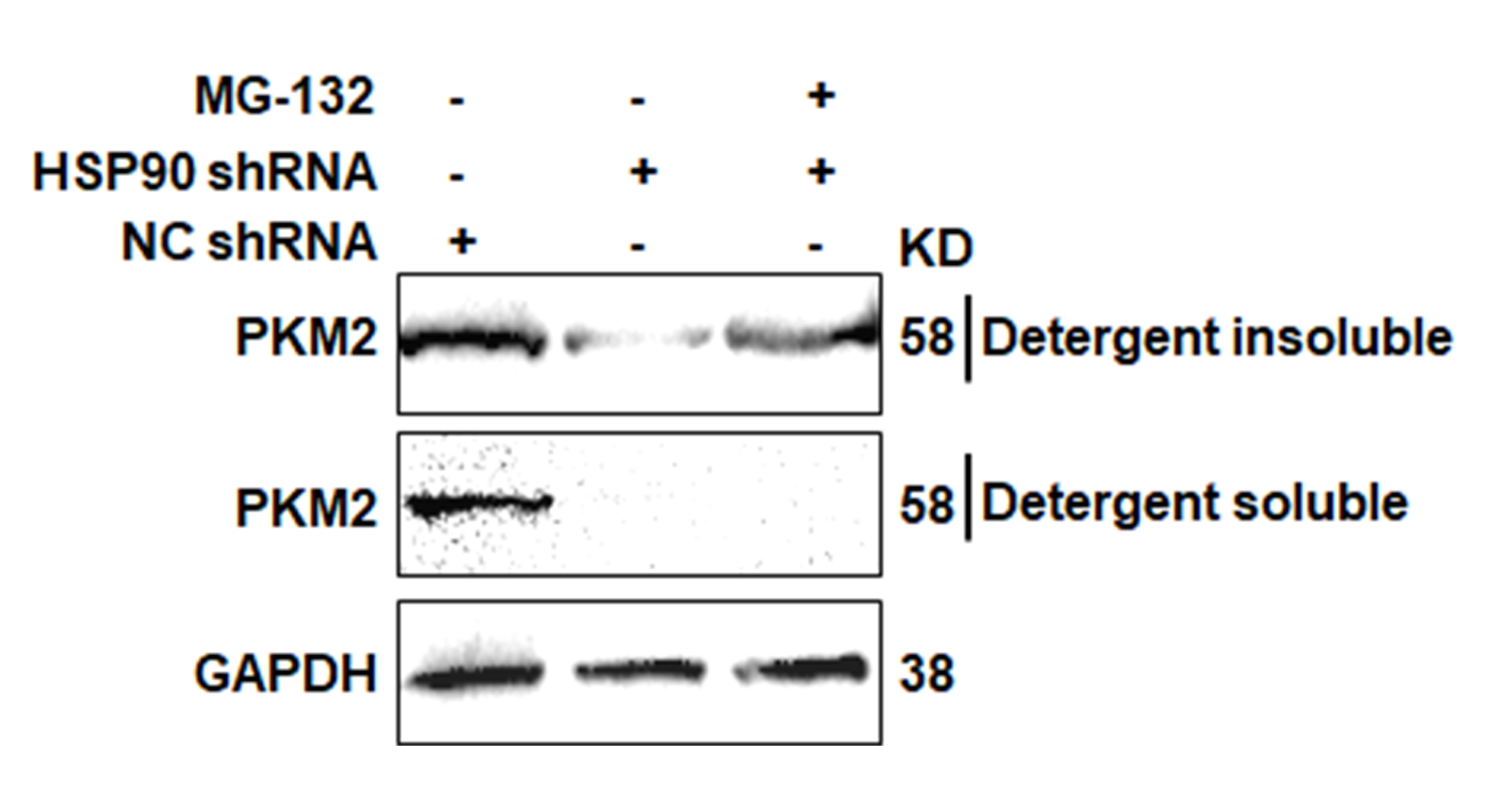

Supplement: Supplementary file 4 — MG132 restored the protein level of PKM2 induced by HSP90 knockdown. MG-132 was used to inhibit the proteasomal degradation in Hep3B cells. MG-132 treatment led to PKM2 accumulation in the detergent insoluble fraction of the cell lysate. (TIFF 7067 kb) [file 12943_2017_748_MOESM4_ESM.tif]

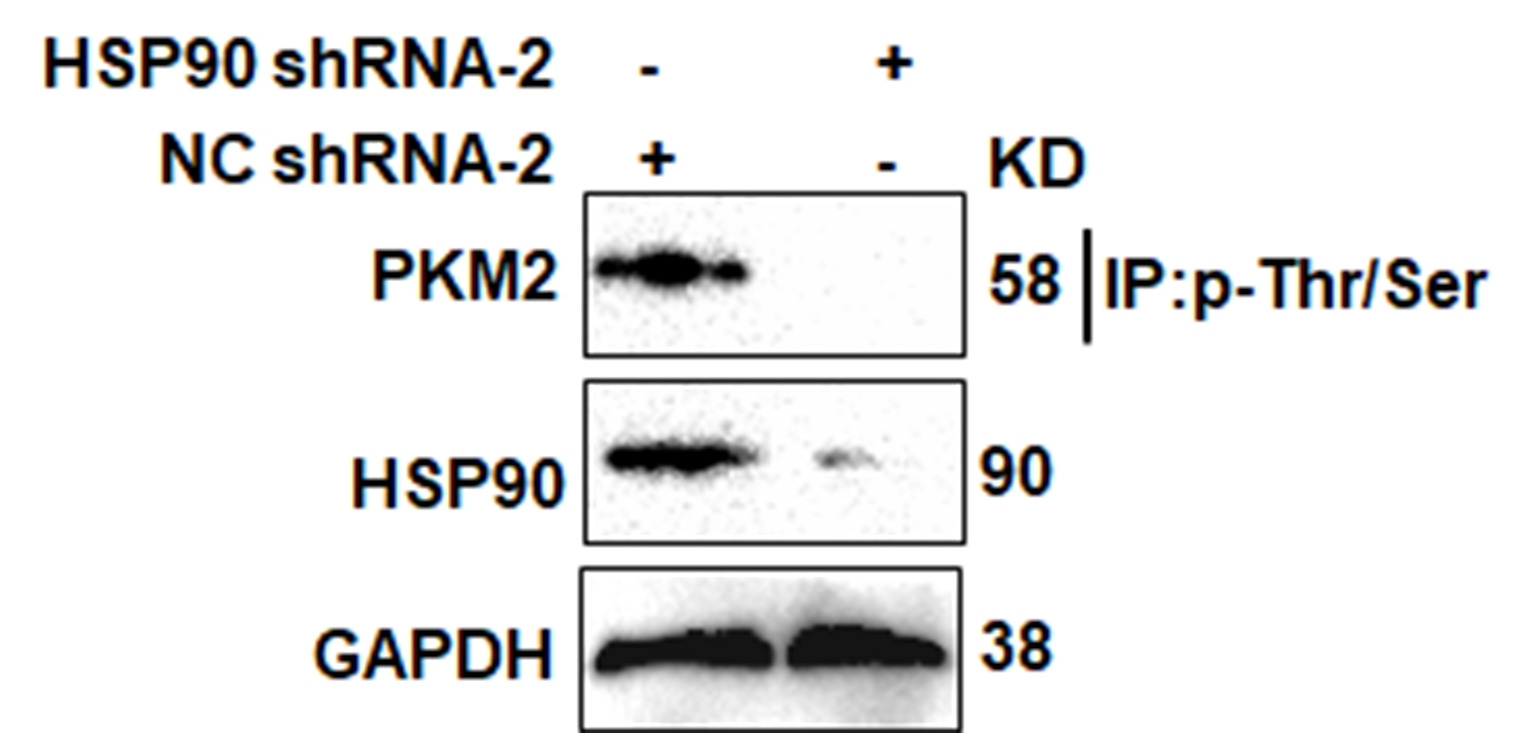

Supplement: Supplementary file 5 — HSP90 knockdown led to decreased Ser/Thr phosphorylation of PKM2. Hep3B cells were transfected with negative control (NC) shRNA or HSP90 shRNA. Representative IP experiments were performed to examine PKM2 Ser/Thr phosphorylation. Total cell lysates were subjected to immunoblotting analysis using specific antibodies against HSP90 and GAPDH. (TIFF 4128 kb) [file 12943_2017_748_MOESM5_ESM.tif]

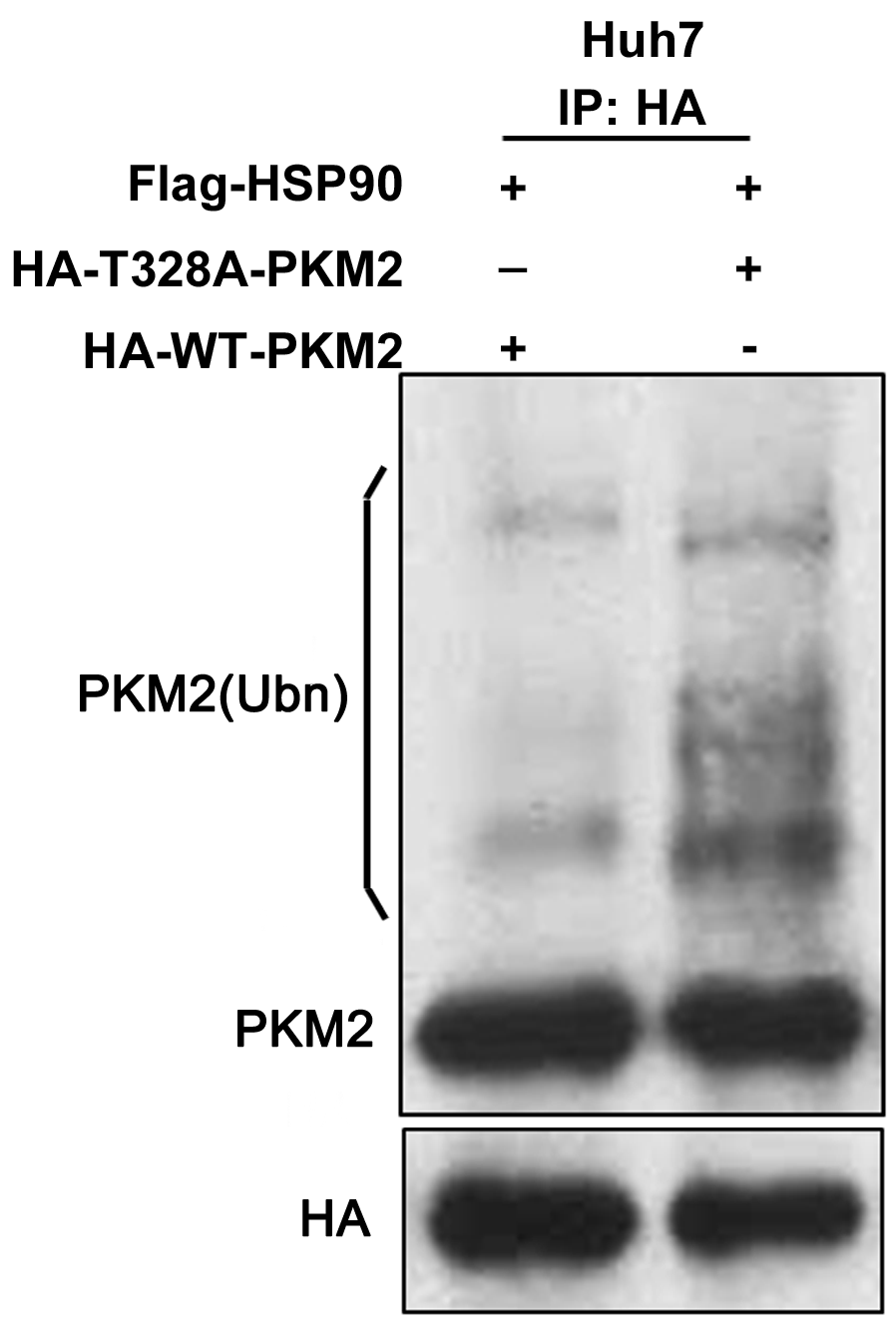

Supplement: Supplementary file 6 — Thr-328 phosphorylation induced by HSP90 decreased the ubiquitination of PKM2 protein. Huh7 cell that were transfected with Flag-HSP90 were then transfected with HA-tagged wild type PKM2 or HA-tagged T328A PKM2. Proteins pull-down by HA antibody was subjected to western blot for ubiquitination. (TIFF 258 kb) [file 12943_2017_748_MOESM6_ESM.tif]

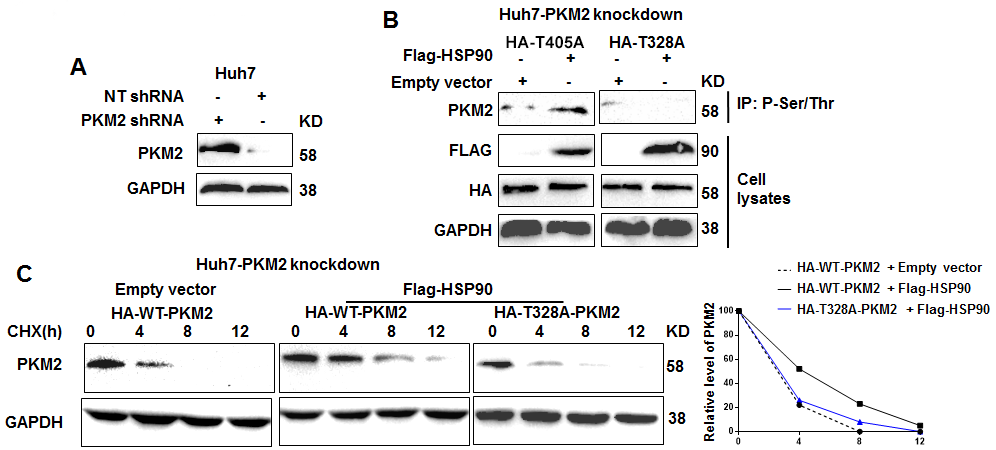

Supplement: Supplementary file 7 — Thr-328 phosphorylation-induced by HSP90 overexpression was critical for maintaining the stability of PKM2. A) PKM2 shRNA was used to knock down exogenous PKM2 in Huh7 cells. PKM2 shRNA effectively depleted the expression of endogenous PKM2 protein. B) Huh7 cells with endogenous PKM2 depleted were transfected with corresponding vectors. T328A mutant, instead of S405A, abrogated the increased phosphorylation of PKM2 induced by HSP90 overexpression. C) Huh7 cells with endogenous PKM2 depleted were co-transfected with corresponding vectors. The protein half-life of HA-tagged WT or mutated PKM2 was analyzed following treatment with cycloheximide (CHX). HSP90 overexpression increased the half-life of the wild type PKM2 while failed to increase the half time of T328A PKM2 mutant. *, P < 0.05 by t test. (TIFF 1330 kb) [file 12943_2017_748_MOESM7_ESM.tif]

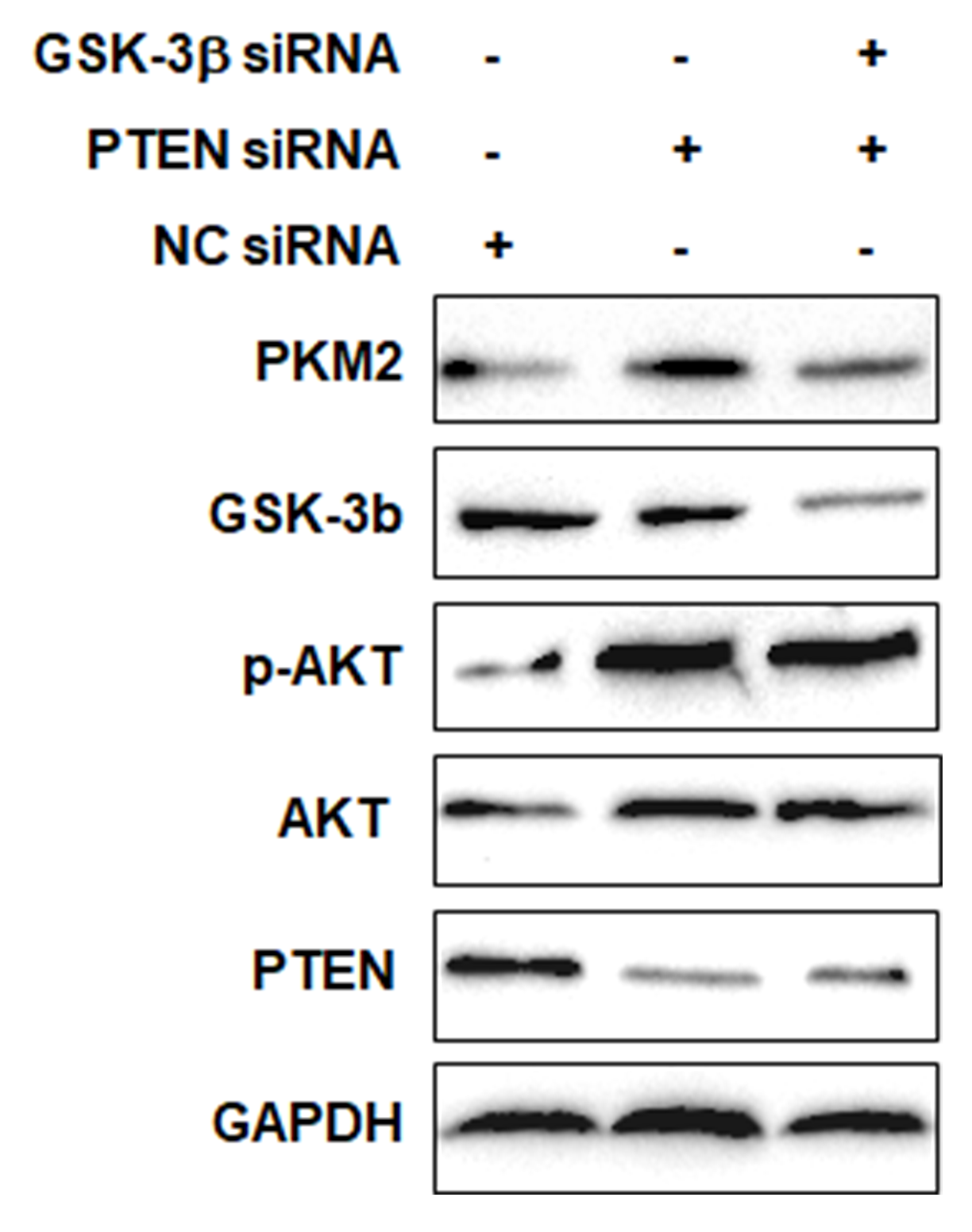

Supplement: Supplementary file 8 — Knockdown of GSK-3β partly inhibited the elevation of PKM2 protein induced by PTEN knockdown. Huh7 cells transfected with PTEN siRNA or control siRNA along with or without GSK-3β. PTEN knockdown, which activated PT3K/AKT pathway, led to increased level of PKM2. Knockdown of GSK-3β partly inhibited the elevation of PKM2 protein induced by PTEN knockdown. (TIFF 6810 kb) [file 12943_2017_748_MOESM8_ESM.tif]

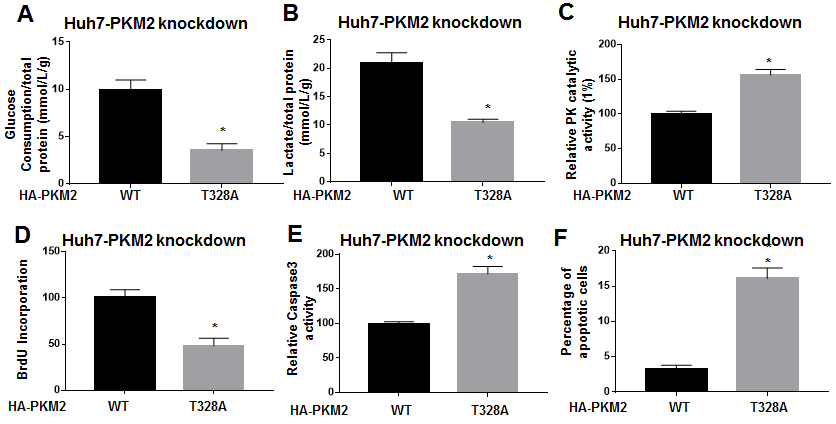

Supplement: Supplementary file 9 — Thr-328 phosphorylation-induced by HSP90 overexpression was critical for maintaining the function of PKM2. In Huh7 cells with endogenous PKM2 depleted, transfection of T328A PKM2 mutant significantly reduced (A) glucose consumption, (B) lactate production and (C) PK catalytic activity of Huh7 cells. Furthermore, transfection of T328A PKM2 mutant significantly reduced (D) proliferation and increased the (E) Caspase-3 activity and (F) apoptosis the of Huh7 cells. (TIFF 1030 kb) [file 12943_2017_748_MOESM9_ESM.tif]

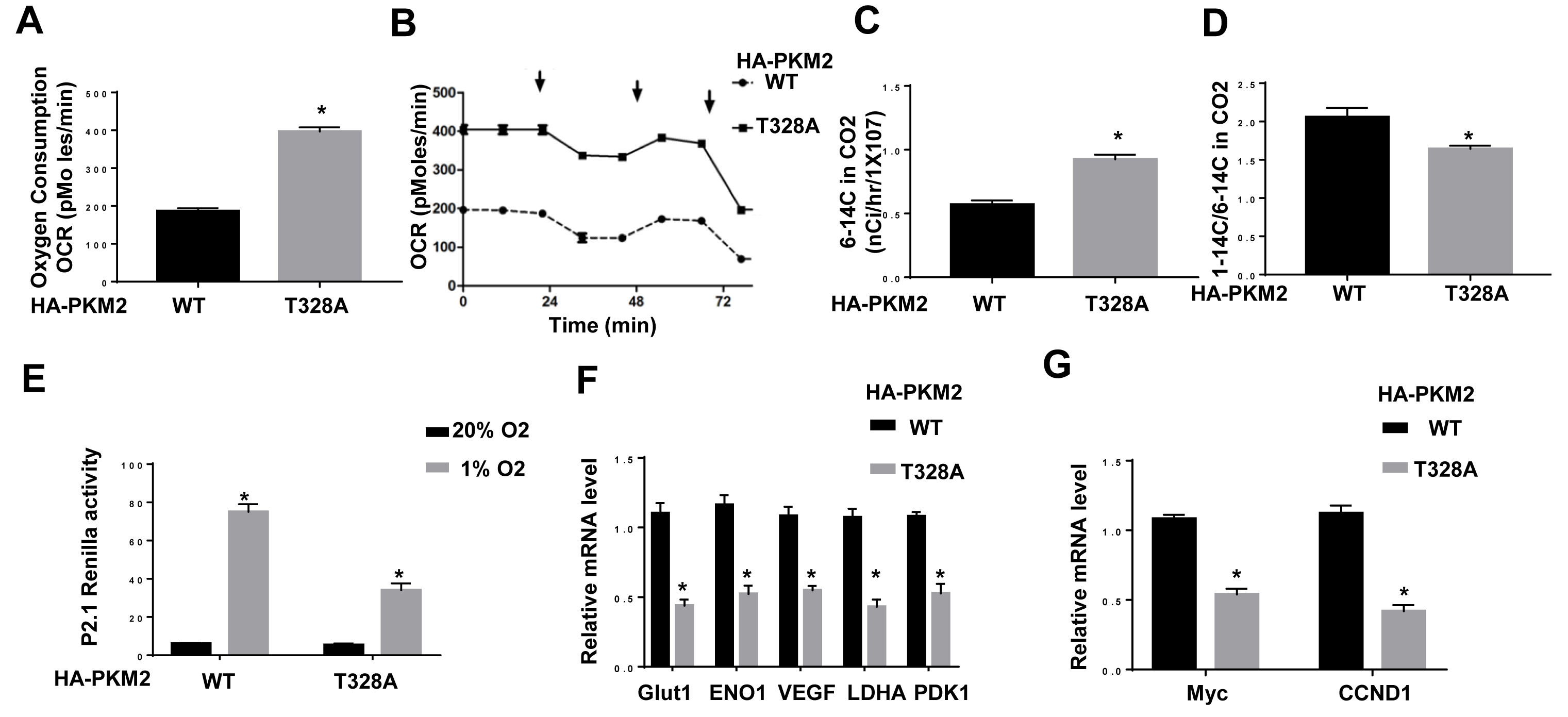

Supplement: Supplementary file 10 — Thr-328 phosphorylation was required for PKM2 to regulate mitochondria respiration and co-factor function. Huh7 cells were transfected with HA-tagged PKM2-WT or T454A. Cells were re-plated into appropriate plates for analysis of O2 consumption, OCR, 6-14CO2 and ratio of 1-14CO2 to 6-14CO2. Compared with Huh7 cells transfected with wild-type PKM2, T328A PKM2 mutant significantly increased (A) O2 consumption, (B) OCR, (C) 6-14CO2 release from [6-14C] glucose while decreased (D) ratio of 1-14CO2 to 6-14CO2. E) HEK293T cells were co-transfected with wild type PKM2 or T328A PKM2 mutant, p2.1 and pSV40-Renilla. Transfected cells were exposed to 20% O2 or 1% O2 for 24 h. The ratio of firefly to renilla luciferase activity was determined. T328A PKM2 mutant significantly reduced the ability of PKM2 to promote hypoxia-induced gene transcription. Huh7 cells were transfected with wild type PKM2 or T328A PKM2 mutant. Two days after transfection, the cells were exposed to 1% O2 or 20% O2 for another 24 h. The mRNA levels of indicated genes were examined by qRT-PCR. T328A pKM2 mutant significantly reduced the ability of PKM2 in activating endogenous (F) HIF-1α target genes and (G) β-catenin target genes. *, P < 0.05 by t test. (TIFF 270 kb) [file 12943_2017_748_MOESM10_ESM.tif]
